# Allele-specific analysis reveals exon- and cell-type-specific regulatory effects of Alzheimer’s disease-associated genetic variants

**DOI:** 10.1038/s41398-022-01913-1

**Published:** 2022-04-18

**Authors:** Liang He, Yury Loika, Alexander M. Kulminski

**Affiliations:** Biodemography of Aging Research Unit, Social Science Research Institute, Duke University, Durham, NC USA

**Keywords:** Genetics, Diseases

## Abstract

Elucidating regulatory effects of Alzheimer’s disease (AD)-associated genetic variants is critical for unraveling their causal pathways and understanding the pathology. However, their cell-type-specific regulatory mechanisms in the brain remain largely unclear. Here, we conducted an analysis of allele-specific expression quantitative trait loci (aseQTLs) for 33 AD-associated variants in four brain regions and seven cell types using ~3000 bulk RNA-seq samples and >0.25 million single nuclei. We first develop a flexible hierarchical Poisson mixed model (HPMM) and demonstrate its superior statistical power to a beta-binomial model achieved by unifying samples in both allelic and genotype-level expression data. Using the HPMM, we identified 24 (~73%) aseQTLs in at least one brain region, including three new eQTLs associated with *CA12*, *CHRNE*, and *CASS4*. Notably, the *APOE* ε4 variant reduces *APOE* expression across all regions, even in AD-unaffected controls. Our results reveal region-dependent and exon-specific effects of multiple aseQTLs, such as rs2093760 with *CR1*, rs7982 with *CLU*, and rs3865444 with *CD33*. In an attempt to pinpoint the cell types responsible for the observed tissue-level aseQTLs using the snRNA-seq data, we detected many aseQTLs in microglia or monocytes associated with immune-related genes, including *HLA-DQB1*, *HLA-DQA2*, *CD33*, *FCER1G*, *MS4A6A*, *SPI1*, and *BIN1*, highlighting the regulatory role of AD-associated variants in the immune response. These findings provide further insights into potential causal pathways and cell types mediating the effects of the AD-associated variants.

## Introduction

Late-onset sporadic Alzheimer’s disease (AD) is the most prevalent progressive neurodegenerative disorder among all dementia cases affecting a large proportion of the elderly population [1]. Late-onset AD (LOAD) etiology is not clearly understood, posing substantive challenges for developing effective intervention and treatment procedures. With the technological breakthrough of next-generation sequencing, large-scale genome-wide association studies (GWAS) over the past decade have hitherto detected >30 genetic loci associated with LOAD [2–9], highlighting the involvement of lipid metabolism and the immune system in the pathogenesis of LOAD. However, except for a few exonic variants in, e.g., *APOE* and *TREM2*, most of these identified signals are in non-coding regions and thus present difficulties in elucidating their causal pathways in neuropathology and pinpointing the genes, cell types, and brain regions mediating their associations with LOAD.

A potential biological consequence of a non-coding genetic variant is local regulation of gene expression. These regulatory variants are called *cis*-acting expression quantitative trait loci (eQTLs). The *cis*-eQTLs have been extensively investigated in multiple tissues and regions across the brain [10–13], substantially improving our understanding of the genetic role of AD-associated variants in local gene regulation. Nevertheless, the statistical power of most transcriptomic studies in the brain is undermined by limited sample sizes and substantial inter-subject noises, including RNA degradation in the post-mortem tissues and heterogeneity of cell-type proportions [11]. More importantly, the brain consists of neural cell types with different morphology. It is largely unclear which cell types mediate the effects of the eQTLs detected at the tissue level. To date, most studies of cell-type-specific eQTLs related to AD have been performed by using an interaction model with deconvolved cell type proportion [11, 14], of which the power and accuracy could be substantially compromised. Fortunately, recent advances in high-throughput single-cell RNA-seq technologies dramatically facilitate the exploration of regulatory events at the single-cell level.

To discover novel eQTLs across more brain regions and further explore their cell-type-specific *cis*-regulatory effects, in this work, we focused on interrogating single nucleotide polymorphisms (SNPs) identified in GWAS of LOAD and conducting an allele-specific eQTL (aseQTL) analysis in four regions in the cerebrum using 3000+ tissue and cell-sorting bulk RNA-seq samples and >0.25 million neural cells from single-nucleus RNA-seq (snRNA-seq) data. We define aseQTLs as genetic variants associated with allelic expression of a local gene. The allelic expression can be measured at a single exonic SNP or a haplotype, and we focused on the SNP-level allelic expression (i.e., SNP-level aseQTLs) in this study. Compared with genotype-based eQTL analysis, aseQTL analysis considerably boosts the statistical power. Allele-specific analysis exploits the imbalance between allelic counts within each of the heterozygous subjects. Because allelic imbalance is insusceptible to inter-subject variation, such as environmental exposures, RNA degradation, and technical noise introduced in experiments, this strategy markedly improves the statistical power and the accuracy of the estimated eQTL effects.

A handful of statistical models have been proposed to leverage allelic imbalance or to detect aseQTLs in bulk RNA-seq data [15–21]. We classify these methods into two major categories. The methods in the first category model the allelic imbalance within each of the heterozygous samples using, e.g., a binomial model [15]. More recent advances in this direction, including a beta-binomial model, a Poisson-Gamma model [20], a logistic mixed-effects model [17], and a binomial generalized linear mixed model [18], further account for overdispersion arising from biological and technical variations. Despite being flexible as these models can be fitted using existing standard statistical tools, they take advantage of only heterozygous samples. The methods in the second category [16, 19] build a dedicated joint likelihood to incorporate the contributions from both allele-specific and genotype-based information. Despite being able to include homozygous samples to improve the statistical power, the second category is less flexible for incorporating interaction or non-linear terms.

Here, we propose a flexible and statistically powerful method based on a hierarchical Poisson mixed model (HPMM) for detecting aseQTLs. The HPMM incorporates both allelic and genotype-level expression and prioritizes the heterozygous samples to boost the statistical power. Our simulation study demonstrates that combining both homozygous and heterozygous samples using the HPMM improves the statistical power compared to a beta-binomial model. Furthermore, it is straightforward to implement using existing statistical tools like the lme4 R package. This is achieved by introducing hierarchical random effects and a coding strategy for genotypes so that allelic counts of a heterozygous sample are remodeled as paired samples. In the snRNA-seq data, unique molecular identifiers (UMIs) are often adopted to mitigate a source of bias introduced in the library amplification step during the sample preparation [22]. Such single-cell data, albeit a more accurate quantification of mRNA molecules, introduce another layer in quantifying the allele-specific expression (ASE). We then further extended this framework of HPMM to accommodate UMI-based snRNA-seq data.

By leveraging the HPMM, we performed a comprehensive aseQTL analysis to investigate the regulatory effects of 33 AD-associated SNPs on the expression of local genes. We examined and compared the aseQTL effects across the prefrontal cortex (PFC), the temporal cortex (TC), the posterior cingulate cortex (PCC), and the head of caudate nucleus (HCN) by using bulk RNA-seq data. The cross-region comparison is of interest because the early stage of LOAD involves the hippocampus and entorhinal cortex, and the degeneration spreads to other brain regions in a later stage, suggesting that the heterogeneity across brain regions may partly explain the pathogenesis of LOAD. To explore the cell-type-specific regulatory mechanisms, we conducted eQTL and aseQTL analyses using snRNA-seq data comprising 240,000+ cells from the PFC. We investigated major neural cell types, including excitatory neurons, inhibitory neurons, astrocytes, oligodendrocytes, and oligodendrocyte progenitor cells (OPCs). To our knowledge, this is the first analysis of aseQTLs for AD at the single-cell level. Because many AD-associated SNPs are located in or near genes specifically expressed in microglia or monocytes (e.g., *TREM2* and *CD33*), we further evaluated the regulatory effects in these two cell types using both cell-sorting bulk RNA-seq and snRNA-seq data. Our results reveal that many AD-associated SNPs are tissue-level aseQTLs in the brain and show exon-specific regulatory effects. Some of these effects are mediated by specific neural or immune cells.

## Results

### Many AD-associated loci are aseQTLs in multiple regions across the cerebrum

**Fig. 1:**
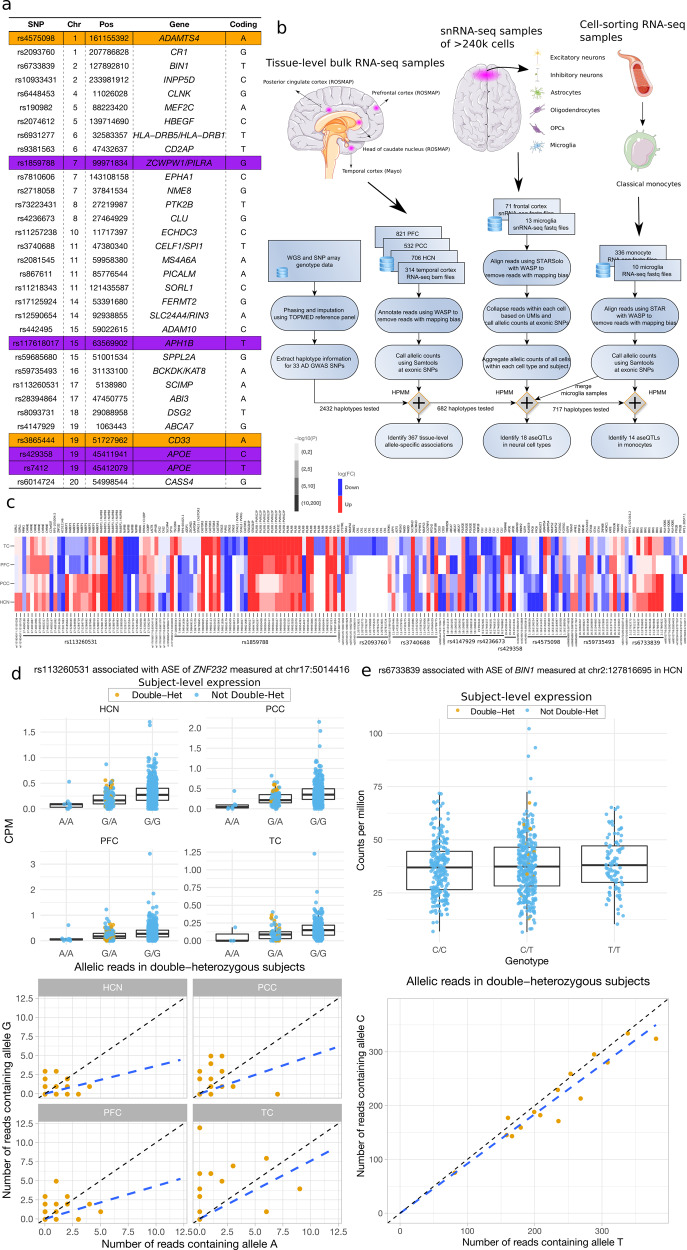
An aseQTL analysis of AD-associated loci in multiple brain regions and cell types. **a** A list of AD-associated SNPs investigated in this study. Pos: the genomic position of the SNP (hg19). Gene: the annotated nearest gene. Coding: the effect allele (coded as 1) corresponding to the effect sizes reported in this study. The UTR and missense variants are highlighted in orange and purple, respectively. **b** An overview of the aseQTL analysis using tissue-level RNA-seq, cell-sorting RNA-seq, and snRNA-seq data sets. The outline includes information about the brain regions, cell types, sample sizes, data processing, and major results. The numbers are the actual sample sizes after removing subjects with missing or inconsistent genotypes. HPMM: the hierarchical Poisson mixed model. **c** Significant aseQTLs and their associated genes identified in the PFC, PCC, HCN, and TC. The genomic position of the exonic variant used for measuring the ASE is shown with the rsID of the AD-associated SNP. log(FC) corresponds to the effect allele given in (**a**). **d** An example showing that the association was mainly determined from the homozygous subjects when the number of double-heterozygous subjects is small. The boxplots summarize the genotype-level expression, and the ASE of the double-heterozygous samples (yellow points) are shown in the scatter plots below. The dashed line is a smooth curve fitted using linear regression. **e** An example of combining evidence from both the ASE and the genotype-level expression to improve the power for detecting the association between the AD-associated SNP rs6733839 and *BIN1*.

We specifically focused on 33 independent (except for the *APOE* ε4 and *APOE* ε2 variants encoded by the minor alleles of rs429358 and rs7412, respectively) AD-associated common SNPs (defined as a minor allele frequency (MAF) > 5%) in the European population reported or replicated in large-scale GWAS and meta-analyses [2–9] (Fig. 1a). Specifically, among the 31 SNPs outside the *APOE* region, 23 SNPs were selected from the lead SNPs reported in [5], one of the latest large-scale GWAS of AD. We further included eight additional variants that were reported as lead SNPs in [7, 8, 23] and were outside the regions of the SNPs selected from [5] (See more details in Table S9). For each of these SNPs, we examined its association with the ASE of proximal genes whose transcription starting site (TSS) is located within a window of ±500k base pairs (bp). We measured SNP-level ASE through exonic SNPs within these local genes across the four brain regions and seven cell types. The pipeline is summarized in Fig. 1b. In the RNA-seq data, we first quantified the ASE based on reads overlapping a heterozygous exonic SNP. In the snRNA-seq data, mRNA molecules with a unique UMI were instead used for the quantification (See Methods). We used WASP [16] to remove reads with potential mapping bias. The exonic SNPs are not necessarily in high linkage disequilibrium (LD) with the AD-associated SNPs, but their haplotype information is available or can be estimated from, e.g., a phasing procedure during imputation. We included only high-quality genotypes to minimize haplotype phasing errors. Using this haplotype information, we employed these exonic SNPs as a proxy for the gene to quantify the ASE associated with each allele of the AD-associated SNP. This SNP-level ASE can only be measured for samples with double-heterozygous genotypes at the GWAS locus and the exonic locus. Our proposed HPMM and coding strategy (See Methods, Fig. S4) build a unified framework to further allow incorporation of subjects that are not double-heterozygous, which improves the statistical power, particularly in situations where the number of double-heterozygous subjects is low. We excluded exonic loci not, or barely overlapping RNA-seq reads (a mean raw count <2) from the analysis of that tissue or cell type because of their low expression and thus the lack of statistical power.

To compare the regulatory effects across different areas in the cerebrum, we performed an aseQTL analysis using four bulk RNA-seq data sets comprising ~2500 samples in the PFC, PCC, and HCN from ROSMAP [24, 25] and in the TC from the MayoRNASeq Project [26] (Fig. 1b). About 51% of the subjects in ROSMAP had samples in all three areas (Fig. S6). The PCC and HCN have not been investigated in the previous studies of *cis*-eQTLs in the brain [12, 13]. We interrogated 2432 haplotypes between the AD-associated SNPs and exonic SNPs within the ±500k bp window. We detected 367 significant (false discovery rate (FDR) adjusted *p* < 0.05) associations (A complete list of these summary statistics is provided in Table S1). These associations involve 24 (72.7%) independent AD GWAS loci in 173 haplotypes, showing differential ASE of 87 genes in at least one of the brain regions (Fig. 1c). While most of these associations showed consistent effects across the four brain regions, some exhibited a region-specific pattern (Fig. 1c). For example, as measured at multiple coding variants, rs113260531-A was associated with reduced expression of *RABEP1* in the PFC and TC but with elevated expression in the other regions (Fig. 1c). rs7982 (chr8:27462481), in almost complete LD with the AD GWAS locus rs4236673, was significantly (*p* = 1E-50) associated with the ASE of *CLU* only in the TC, but not even nominally significant in the other regions (Fig. 1c, Table S1). In addition, rs2093760 and rs442495 were strong aseQTLs of *CR1* and *ADAM10* in the TC, respectively, while the expression of both genes was hardly detectable in the other regions. These observations suggest that the regulatory effects of certain AD-associated SNPs are region-dependent.

We showed in the simulation study (Fig. S7) that, by combining both allelic and genotype-level expression, the proposed HPMM is statistically more powerful than the beta-binomial model. The advantage of the HPMM is evident in some of these findings. For example, the association between rs113260531 and *ZNF232* became significant after combining evidence primarily from the genotype-based association, although the evidence for the allelic imbalance was equivocal due to the small number of double-heterozygous samples (Fig. 1d). This demonstrates the advantage of incorporating subjects that are not double-heterozygous, particularly when one SNP has a low MAF. Similarly, the significance of the association between rs6733839 and *BIN1* was achieved by combining the weak evidence from both genotype-level and allelic expression (Fig. 1e).

### Analysis of aseQTLs reveals exon-specific associations in *CD33* and *APOE*

The SNP-level ASE facilitates the exploration of exon-specific aseQTLs compared to haplotype-level ASE, which aggregates reads across all heterozygous loci within a gene. Opposite effects or weaker significance observed at exonic SNPs in different exons within the same gene might suggest that the identified aseQTLs are splicing variants or associated with differential transcript expression. Weaker significance at some exonic variants might also be due to lower exon expression or fewer double-heterozygous subjects. Among the significant associations, we observed exon-specific effects at two exonic loci (chr19:51728477 and chr19:51728641) in the second exon of *CD33* (FDR *p* < 0.05), and the effects observed in the other exons were not significant (Table S1). Because *CD33* is predominantly expressed in microglia among the neural cells [27, 28], this region-specific result supports the previous finding of rs3865444 or its haplotype as a splicing variant of exon 2 of *CD33* in microglia [29].

**Fig. 2:**
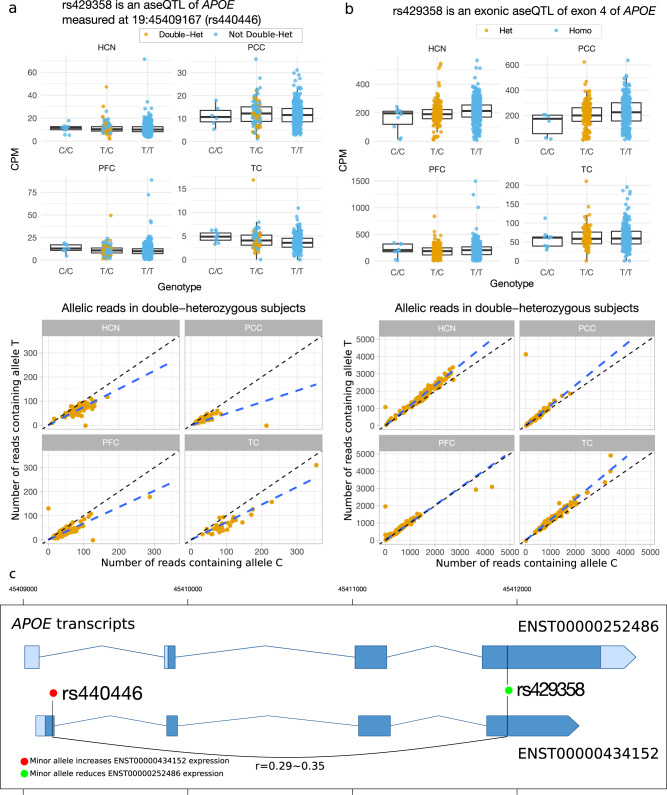
The *APOE* ε4 variant rs429358-C shows exon-dependent association with the ASE of *APOE*. **a** rs429358-C is associated with increased expression of exon 2 measured at the exonic variant rs440446. **b** rs429358-C is associated with increased expression of exon 4 measured at itself. In both (**a**) and (**b**), the evidence from the genotype-level expression and the ASE is consistent. **c** The exon-dependent associations are due to the LD between rs429358 and rs440446, which had opposite effects on different *APOE* transcripts. The genomic coordinates are based on the human assembly hg19.

Additionally, we observed exon-dependent associations in, e.g., *APOE*, *CLU*, and *PILRB.* Allelic associations in these genes exhibit opposite effects at SNPs located in different exons (Fig. 1c). Further scrutiny of these opposite effects reveals that these exon-dependent associations result from the fact that the AD GWAS SNPs are in high LD with multiple exonic variants that showed opposite effects on different transcripts (Table S1). For example, we observed that the *APOE* ε4 allele (rs429358-C), the leading genetic risk factor, was associated with elevated expression of exon 2 measured at rs440446 (Fig. 2a), but with reduced expression of exon 4 of *APOE* (Fig. 2b). These effects were consistent across multiple regions in both homozygous and double-heterozygous subjects (Fig. 2a, b). A further investigation reveals that the *APOE* ε4 variant is in mild LD with rs440446, which is located in another transcript (ENST00000434152) of *APOE* (Fig. 2c). The expression of ENST00000434152 is much lower than that of the major transcript (ENST00000252486) (Fig. 2a, b), probably due to its premature termination (Fig. 2c), and ENST00000434152 does not lead to a truncated ApoE protein [30]. As ENST00000252486 accounts for the vast majority of the *APOE* expression, the imbalanced ASE indicates that the haplotype containing the *APOE* ε4 allele has a repressive effect on the ApoE ε4 isoform. We then attempted to elucidate whether the association between the *APOE* ε4 allele and the decreased *APOE* expression was mediated by a higher frequency of AD patients in the *APOE* ε4 carriers. We carried out an allelic analysis using the healthy controls alone in the PFC, PCC, and HCN (The cohort in the TC has only a small number of control subjects and thus was not investigated). We found that the significant associations were preserved in the control subjects, suggesting that the regulatory effects of the *APOE* ε4 allele were not mediated through the diagnosis of AD.

### Interpretation of the identified allelic associations

**Fig. 3:**
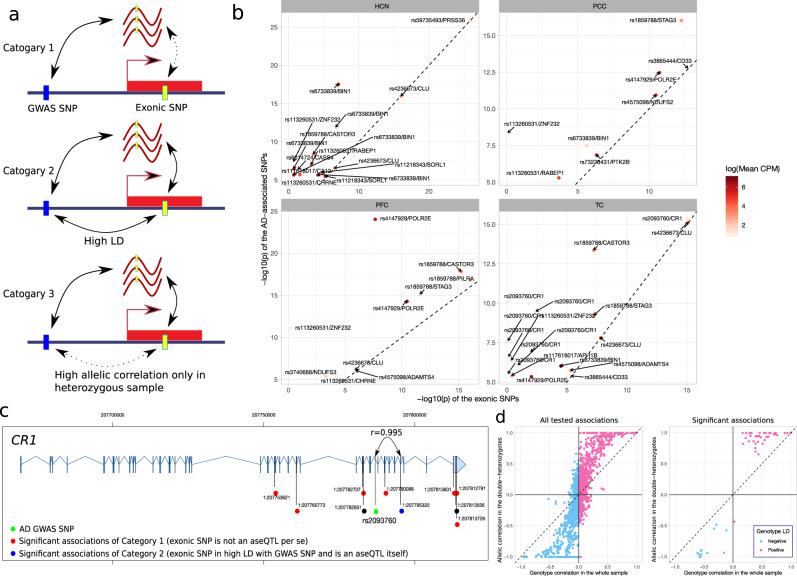
Interpretation of the identified aseQTLs in the PFC, PCC, HCN, and TC. **a** Classification of the identified associations into three categories based on the LD between the AD-associated SNP and the exonic SNP and whether the AD-associated SNP is more significantly associated with the ASE than the exonic SNP. **b** Comparison of the p-values between the AD-associated SNP (y-axis) and the exonic variant (x-axis) for the significant aseQTLs in the first category. The color of the points indicates the expression level measured at the exonic variant. The labels include the AD-associated SNP and its associated gene. For better visualization, we omit one significant association between rs4236673 and *CLU* in the TC with a *p* value < 1E-50. **c** Exon-dependent aseQTLs identified in *CR1*. Green: the AD-associated SNP. Blue: the significant association classified as the second category. Red: significant associations classified as the first category. Black: Nonsignificant associations. The genomic coordinates are based on the human assembly hg19. **d** Comparison of the genotype correlation between the AD-associated and the exonic SNPs in the whole sample (x-axis) and the allelic correlation between the two SNPs observed in double-heterozygous samples (y-axis). The difference between these two correlations is more noticeable in the identified significant aseQTLs.

As shown in the example of *APOE*, the identified aseQTLs can be mediated by their high LD with the exonic SNPs, which might be the driving variant per se. Therefore, to further explore the biological interpretation, we divided these significant associations into three categories based on the LD between the SNPs and whether the exonic SNP per se was associated with the ASE (Fig. 3a). This strategy attempted to evaluate whether the detected allelic imbalance was driven by the AD-associated SNP or the exonic proxy SNP. The first category includes the situation in which the *p* value of the exonic SNP is less significant than that of the AD-associated SNP for the association with the ASE (Fig. 3a, Top). We found 53 associations involving 19 genes in this category (Table S1, Fig. 3b). These associations are likely not mediated by the exonic SNP because of its less significant *p* value. The allelic imbalance arises from either the regulatory effects of the GWAS SNP or the effect of another exonic variant in high LD with the GWAS SNP. A notable example is those associations between rs2093760 and *CR1* in the TC. The AD-associated SNP rs2093760 was significantly associated with the ASE of multiple exons of *CR1*, but almost all exonic variants were not or were weakly associated with the ASE. The only exception is the missense variant rs2296160, which was in nearly complete LD (*r* = 0.995) with rs2093760 and very strongly (*p* = 8.95E-16) associated with the ASE (Table S1, Fig. 3c). This suggests that the missense variant rs2296160 likely mediates the associations between rs2093760 and the ASE of *CR1*. This situation is described in the second category, in which the *p* value of the GWAS SNP is less significant than that of the exonic SNP, but the two SNPs are in high LD (defined by |*r* | >0.8) (Fig. 3a, Middle). We found 17 other associations in this category, including rs4236673 with *CLU*, rs4575098 with *ADAMTS4*, rs4575098 with *B4GALT3*, rs59735493 with *PRSS36*, rs3865444 with *CD33*, and rs442495 with *ADAM10* (Table S1). These AD GWAS SNPs were in almost complete LD with the exonic SNPs used for measuring the ASE. Hence, the effect can be driven by the disease-associated SNP, an exonic SNP in high LD with it, or a haplotype harboring alleles of these two SNPs. It is not straightforward to distinguish the causal variant without combining additional annotation information or more samples.

We classify the remaining significant aseQTLs as the third category, mainly including the situation in which the disease-associated SNP was not in strong LD with the exonic SNP, and the association between the ASE and the exonic SNP was much more significant than that of the AD-associated SNP (Fig. 3a, Bottom). In this category, the allelic imbalance was probably driven by the exonic SNP, and the observed association between the ASE and the AD-associated SNP often resulted from a large correlation between the alleles of the two SNPs in the double-heterozygous subjects. As shown in Fig. 3d, although two SNPs are in very mild LD, their conditional correlation in the double-heterozygous samples can be much larger, particularly in those significant associations. Compared to a general eQTL analysis, this inflated allelic correlation among the double-heterozygous subjects plays a prominent role in confounding the observed association between the ASE and the disease-associated SNP. Moreover, we observed an even more pronounced difference between the allelic correlation in the double-heterozygous subjects and the genotype correlation in the whole sample when the SNPs have a low MAF (Fig. S5) or the sample size is small (See Methods for more details). Therefore, LD has a prominent influence on detecting SNP-level aseQTLs, and it should be cautious about interpreting the associations in this category.

To further assess these significant associations, we compared the identified aseQTLs with gene-level eQTLs reported from a previous meta-analysis using the same cohorts in the PFC and TC [12] and previous eQTL analyses in ROSMAP [31], GTEx (v8) [13] (PFC, hippocampus, and anterior cingulate cortex (ACC)), CommonMind [32], and BrainSeq [33]. Among the aseQTLs in the first and second categories, the associations with *NDUFS2, ADAM10, CR1, APH1B, POLR2E, ZNF232, PILRA, STAG3*, and *PRSS36* are reported in at least one of these studies. The association between rs2093760 and *CR1* was identified only in the TC in [12] and in the hippocampus in GTEx, corroborating our finding of its region-specific effects. However, our detected aseQTLs associated with *CLU*, *BIN1*, *CD33, PTK2B, B4GALT3, CASTOR3*, and *ADAMTS4* in the brain are not reported in these gene-level results, probably because exonic SNPs disrupting the exon-level or transcript-level expression might not be detected from the analysis of the gene-level expression. Therefore, we then compared the summary statistics of splicing QTLs (sQTLs), transcript-level and exon-level eQTL analyses in GTEx [13], CommonMind [32], BrainSeq [33], and ROSMAP [34]. Indeed, we found that many of these aseQTLs are exon-level or transcript-level eQTLs. The exonic SNPs used to measure the ASE with *CLU*, *PTK2B*, *BIN1*, and *CASTOR3* are in high LD with the sQTLs in the PFC reported in [34]. Notably, for *CLU*, our aseQTL analysis captured two sQTLs, rs9331888, reported in [34, 35] and rs7982, reported being associated with intron retention in exon 5 [36]. The AD GWAS SNP rs4236673 is in almost complete LD with rs7982 and mild LD with rs9331888 (Table S1). In addition to these consistent findings, the associations between rs117618017 and *CA12* in the HCN, rs113260531 and *CHRNE* in the PFC and HCN, rs6014724 and *CASS4* in the HCN, are novel.

### No evidence of interaction between aseQTLs and age, sex, or AD

Because age is one of the major risk factors of LOAD and has been reported to regulate the expression of microglial genes related to cytoskeleton, immune response, and cell adhesion [37], we then investigated whether these aseQTL effects were different across age groups in the four brain regions. We transformed age into a dichotomized variable (age at death ≤85 or >85). We carried out an interaction analysis by adding the dichotomized age variable and its interaction term with the genotype to the HPMM. We restricted our interaction analysis to the 367 significant associations identified in the region-specific aseQTL analysis. We tested the null hypotheses of no interaction effect on the ASE between an aseQTL and age; that is, the impact of the aseQTL on the ASE is homogeneous across the age groups. We did not find significant interaction effects of age after adjusting for multiple testing (FDR *p* < 0.05) (Table S2). In addition, we found no interaction effects between the detected aseQTLs and sex or the diagnosis of LOAD.

### Cell-type-specific analysis reveals that rs2093760 is an eQTL of *CR1* in oligodendrocytes

Given the evidence that many AD GWAS SNPs were aseQTLs in the cortex, we next attempted to pinpoint the neural cell types that mediate such associations. We collected snRNA-seq data in the frontal cortex comprising >240,000 cells from 71 subjects from two cohorts in ROSMAP [27, 38] (Fig. 1b). Using the cell type annotation provided in [27, 38], we classified these cells into six major neural cell types: excitatory neurons, inhibitory neurons, astrocytes, microglia, oligodendrocytes, and OPCs. Unlike the bulk RNA-seq data, UMIs are utilized in these snRNA-seq data sets to mitigate the amplification bias. Since multiple reads tagging with the same UMI originate from the same mRNA molecule, the UMI-based snRNA-seq data introduce another layer of complication in the allele-specific analysis. We, therefore, called the ASE at the UMI level by collapsing all reads that share a similar UMI barcode (see the Methods section for more details). We carried out a cell-type-specific aseQTL analysis for the AD-associated SNPs in each cell type using the UMI-level ASE. We quantified the ASE at exonic SNPs in local genes whose TSS is located within a window of ±500k bp surrounding the AD-associated SNPs.

**Fig. 4:**
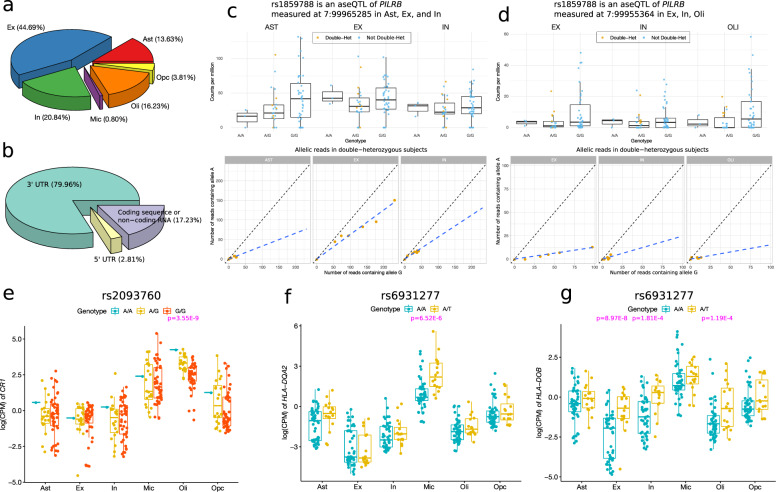
Cell-type-specific aseQTL and *cis*-eQTL analysis using snRNA-seq data in the frontal cortex. **a** Percentage of the six neural cell types among the examined associations. **b** The location-based annotation (3’ UTR, 5’ UTR, or coding regions) of the exonic SNPs used in the aseQTL analysis with their percentage. **c** and **d** rs1859788 is a significant aseQTL of *PILRB* measured at two exonic loci in multiple neural cell types. The boxplots summarize the genotype-level expression, and the ASE of the double-heterozygous samples (yellow points) are shown in the scatter plot below. The dashed line is a smooth curve fitted using linear regression. **e**–**g** Three significant genotype-based cell-type-specific *cis*-eQTLs identified using a pseudo-bulk sample by aggregating cells in the snRNA-seq data. The *p* values of the significant associations are highlighted in pink.

We tested 499 associations between 28 AD-associated SNPs and 118 local genes that showed at least moderate expression (mean count>2), among which 327 tests (65.5%) were in the two types of neurons (Fig. 4a). Only four of these tests were in microglia because of the tiny proportion of microglia in the entire library of the snRNA-seq data. Compared to the bulk RNA-seq data, we observed substantially attenuated expression in the 5’-end, which is expected because of the bias of read coverage toward the 3’-end in the 3’-sequencing snRNA-seq data. Consequently, the reads in the other genomic regions are often not sufficiently abundant for measuring the ASE. Therefore, most exonic variants under evaluation were located in the 3’ untranslated region (UTR) (Fig. 4b). We identified 11 significant associations (FDR *p* < 0.05) between two AD-associated SNPs and the ASE of three genes, including *PILRB*, *TRIM4*, and *MTCH2* (Table S3). All these associations were detected in the region-specific analysis (Fig. 1c). Specifically, the AD GWAS SNP rs1859788 was associated with the allelic imbalance of *PILRB* in excitatory and inhibitory neurons, astrocytes, and oligodendrocytes. The trend of the genotype-level expression in the homozygous subjects is concordant with the imbalance of the ASE measured at two exonic variants (chr7:99965285 and chr7:99955364) in *PILRB* (Fig. 4c, d). These associations belong to the third category described above. rs1859788 was in complete LD with the two exonic SNPs only in the double-heterozygous subjects, but the correlation was low in the whole sample (*r* = ~0.2). Additionally, both exonic SNPs were much more strongly associated with the allelic imbalance of *PILRB* (Table S3). Therefore, these associations are likely mediated by the two exonic SNPs and not driven by the regulatory effects of rs1859788. Similar situations were observed for the other two associations between rs1859788 and *TRIM4*, and rs3740688 and *MTCH2* in excitatory neurons (Table S3, Fig. S3). To further assess these identified associations, we performed a general genotype-level eQTL analysis for rs1859788 and rs3740688. Indeed, these SNPs were not significant in the general eQTL analysis (Table S4), suggesting that the observed associations in the allelic analysis are likely due to their high LD with the exonic SNPs in the double-heterozygous subjects.

A drawback of the allelic analysis using the snRNA-seq data is the low expression called at exonic variants that are not close to 3’-end, which potentially misses many signals and leads to a small number of identified associations. To compare the results more thoroughly between the allele-specific and the genotype-level eQTL analyses, we also performed a genotype-level *cis*-eQTL analysis for local genes within a ± 500k bp window in each of the six neural cell types. We identified three significant associations (FDR p < 0.05) (Table S4), including an association between rs2093760 and *CR1* in oligodendrocytes (Fig. 4e). *CR1* was abundantly expressed only in oligodendrocytes and microglia (Fig. 4e). Because no association was detected in microglia, the tissue-level association between rs2093760 and *CR1* (Fig. 1c) is likely attributed to its effect in oligodendrocytes. We also found associations between rs6931277 and *HLA-DOB* and *HLA-DQA2* in excitatory neurons and microglia, respectively (Fig. 4f, g). The association between rs6931277 and *HLA-DOB* was also nominally (*p* < 0.05) significant in inhibitory neurons and oligodendrocytes.

### Multiple AD-associated SNPs are aseQTLs in microglia

**Fig. 5:**
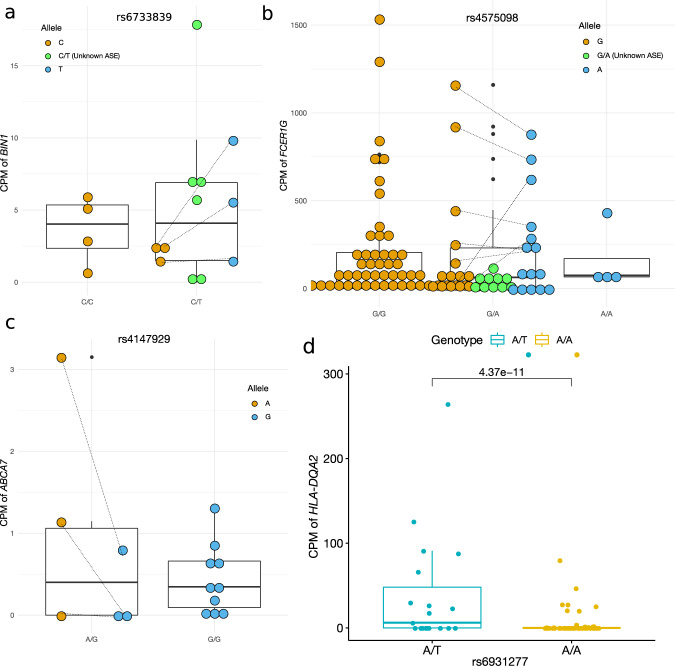
Cell-type-specific aseQTL and *cis*-eQTL analyses in microglia using combined snRNA-seq and cell-sorting bulk RNA-seq data sets. **a**–**c** Three significant aseQTLs identified in microglia, which are also significant in the tissue-level aseQTL analysis. Unknown ASE: The ASE of these subjects is not available because they are not double-heterozygous. The pair of the allelic counts of a double-heterozygous subject are connected by a dashed line. Some lines are omitted from the plot of *FCER1G* to improve visibility. The CPM of the ASE of the double-heterozygous subjects is normalized by half of its total library size. **d** rs6931277 is a significant genotype-level *cis*-eQTL of *HLA-DQA2* in microglia.

Many AD-associated loci are located in genes that are exclusively or abundantly expressed in microglia. Unfortunately, microglia accounted for only a tiny fraction of the total cell population in the two snRNA-seq data sets in the frontal cortex. To increase the statistical power to detect aseQTLs in microglia, in addition to the 71 snRNA-seq samples in the frontal cortex, we further added seven samples from a microglia-specific snRNA-seq data set [39] and ten samples from a cell-sorting bulk RNA-seq data set [40]. We tested 183 associations between the imbalance of ASE and haplotypes in which there were at least three double-heterozygous samples. We identified seven significant associations (FDR *p* < 0.05) (Table S5) involving six genes (*BIN1*, *ITGAM*, *FCER1G*, *MS4A7*, *ATXN3*, and *ABCA7*). The associations with *BIN1, FCER1G*, and *ABCA7* were also observed in our region-specific aseQTL analysis (Fig. 1c), suggesting that some of these tissue-level associations might be driven by their effects in microglia. For example, because microglia are the primary cell type expressing *FCER1G*, its tissue-level association is likely mediated through microglia. For these three genes, the ASE and the genotype-level expression exhibit consistent trends (Fig. 5a–c). The other three genes (*ITGAM, MS4A7*, and *ATXN3*) were not detected in the region-specific analysis, probably due to their much lower expression at the tissue level. Most of the significant associations in microglia were primarily driven by strong allelic imbalance observed in the double-heterozygous samples (Fig. 5a, c, Fig. S2). Because of the small sample size, the allelic imbalance can be attributed to an inflated correlation between the AD-associated SNP and the exonic SNP in the double-heterozygous subjects. Further study with larger sample size is required to confirm these findings.

To compare with the allele-specific analysis, we also performed a genotype-level eQTL analysis using these 88 samples, including the 17 microglia-specific samples and the 71 samples comprising cells annotated as microglia in the two snRNA-seq data sets in the frontal cortex. Our analysis confirmed that rs6931277 was a *cis*-eQTL of *HLA-DQA2* in microglia with a more significant *p* value after including the microglia-specific samples (Fig. 5d, Table S6). In addition, among the top five genes, we observed associations between rs4575098 and *ADAMTS4* (*p* = 7.4E-4) and between rs867611 and *PICALM* (*p* = 9E-4) (Table S6). In contrast to the oligodendrocytes, rs2093760 was not associated with the expression of *CR1* in microglia. These associations were preserved after adjusting for the AD diagnosis (Table S6), suggesting that these cis-regulatory effects are not affected by the disease status.

### Identification of aseQTLs with immune-related genes in monocytes

Multiple putative AD-related genes are also abundantly expressed in monocytes, implying that monocytes might also be involved in AD by, for example, infiltration through the blood-brain barrier [41–43] or its breakdown [44, 45]. We next investigated the association between the AD-associated loci and the ASE in CD14^+^CD16^-^ classical monocytes in a cohort [40] comprising >600 cell-sorting RNA-seq samples from ROSMAP (Fig. 1b). We identified 14 significant associations (FDR *p* < 0.05) involving six AD-associated SNPs and nine genes (Table S7). Most of these genes are related to the immune system, including *CD33*, *MS4A6A*, *SPI1*, *HLA-DQB1*, and *FCER1G*. In these associations, the GWAS loci were in moderate or high LD ( | *r* | >0.5) with the exonic variants.

**Fig. 6:**
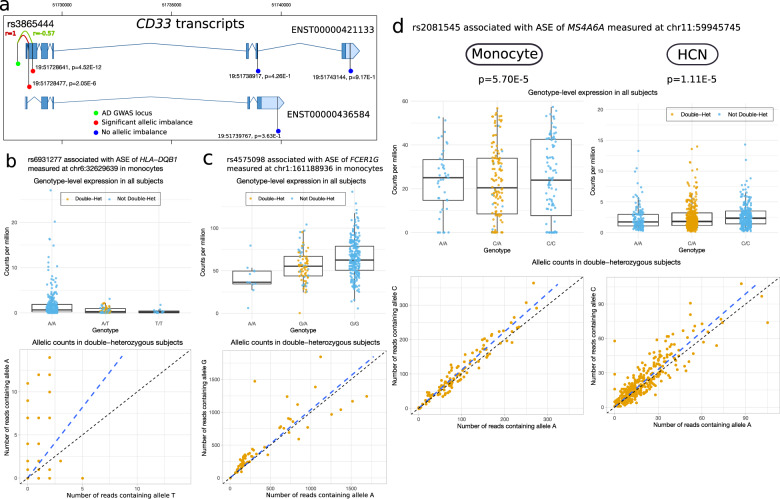
The aseQTLs identified in the classical monocytes, which are also identified in neural cell types or brain regions. **a** Exon-specific aseQTLs of *CD33* identified in monocytes. Green: the AD-associated SNP rs3865444. Red: exonic loci with a significant FDR p-value (FDR *p* < 0.05). Blue: exonic loci with a nonsignificant *p* value (*p* > 0.05). The red and green curves indicate complete and moderate LD between rs3865444 and the two exonic variants in exon 2 of *CD33*. *P* values: the *p* values of the association between rs3865444 and the ASE measured at the exonic locus. The genomic coordinates are based on the human assembly hg19. **b** rs6931277 is a significant aseQTL of *HLA-DQB1* in the monocytes. **c** rs4575098 is a significant aseQTL of *FCER1G* in the monocytes. **d** rs2081545 is a significant (FDR *p* < 0.05) aseQTL of *MS4A6A* in monocytes, which is consistent with its *p* value and effect in the HCN (FDR *p* < 0.1). The boxplots summarize the genotype-level expression, and the ASE of the double-heterozygous samples (yellow points) are shown in the scatter plot. The dashed line is a smooth curve fitted using linear regression.

Similar to the results in the region-specific analysis, rs3865444 was associated with the ASE of the second exon of *CD33* measured at chr19:51728641 (rs2455069) and chr19:51728477 (rs12459419) in monocytes (*p* = 4.52E-12 and 2.05E-6) (Fig. 6a), confirming that this AD-associated SNP is an sQTL of exon 2 reported in [46]. Our follow-up transcript-level eQTL analysis also shows that rs3865444 was only associated with two transcripts (ENST00000421133 and ENST00000601785) (Table S8) that overlap the second exon among all seven transcripts of *CD33*.

In addition, many associations showed consistent effects compared to those detected in microglia or the brain regions. For example, rs6931277 was associated with the ASE of *HLA-DQB1* in monocytes (Fig. 6b). It was also associated with *HLA-DQB1* and *HLA-DQA2* in microglia (Tables S4, S5) and with *HLA-DOB* in neurons (Table S4). Moreover, rs6931277 is an sQTL of multiple Class II HLA genes in almost all tissues in GTEx [13]. This suggests that rs6931277 may affect different genes in a cell-type-dependent manner. As another example, rs4575098, an AD-associated SNP located in the UTR of *ADAMTS4*, was in moderate LD (*r* = 0.59) with an exonic variant rs11421, located in the 5’ UTR of *FCER1G*, which was itself a significant aseQTL of *FCER1G* (Fig. 6c). This association was also consistent with those detected in the region-specific analysis and in microglia in terms of the direction of the effect (Table S1, Table S5). Likewise, the association between rs2081545 and the ASE of *MS4A6A* was observed in both monocytes and the HCN (FDR *p* < 0.1), and the direction of effect sizes was consistent (Table S1, Fig. 6d). The association between rs2081545 and *MS4A6A* was observed in two of the exons measured at rs7232 and rs12453. The lack of significance in the other exons was probably due to their much lower expression (Table S7).

The evidence from allelic imbalance is in line with genotype-level differential expression in most of these associations (Fig. 6b–d, Fig. S1). Allelic imbalance plays a more crucial role in determining the association when the conclusion cannot be drawn from genotype-level expression alone (e.g., *MS4A6A* and *HBEGF*). In comparison, a previous eQTL analysis in classical monocytes using the DICE (~100 samples) [47] identifies associations with four SNPs, one of which between rs6931277 and *HLA-DQB1* was also observed in this analysis. This study did not investigate the other associations because these genes were not within our defined window for a *cis*-gene.

## Discussion

In this study, we investigated the regulatory effects of AD GWAS SNPs using the allelic expression in four brain regions, six major neural cell types, and monocytes. Our results replicate not only previously reported signals and, most importantly, also discover novel aseQTLs. It is shown in [12] that the regulatory patterns of eQTLs are different between the cortex and the cerebellum. Our SNP-level aseQTL analysis further reveals that the regulatory effects of AD-associated SNPs can be heterogeneous across different brain regions and neural cell types, even within the cortex, and can be exon-specific.

We have demonstrated that the SNP-level aseQTL analysis not only identifies eQTLs but can also uncover exon-specific regulation. As an example, our aseQTL analysis of *CD33* detected significant associations between rs3865444 and allelic imbalance at exonic SNPs exclusively in exon 2. These associations were observed in monocytes and multiple brain regions, including the TC, PCC, and HCN, supporting previous findings showing that the AD GWAS locus rs3865444 is associated with the splicing of exon 2 of *CD33* [29, 34, 46]. The almost complete LD between the promoter variant rs3865444 and the exonic variant rs12459419 makes it challenging to determine the causal variant. Another example is *CLU*, of which our aseQTL analysis captured two sQTLs in the promoter and exon 5. A recent study reveals that rs7982 is associated with intron retention in three regions in the temporal lobe, including TC, superior temporal gyrus, and parahippocampal gyrus [36]. Our result shows no association in the PFC, PCC, or HCN, suggesting that this sQTL is region-dependent.

Interestingly, the *APOE* ε4 variant is associated with attenuated expression of the major transcript of *APOE* based on the evidence from both allelic imbalance and gene-level expression. These results corroborate previous findings of lower mRNA and protein levels of *APOE* in ε4 carriers [48–51]. Our findings reveal that this association remains significant in the control subjects and is across all these brain regions. The expression of the ApoE ε4 isoform is lower than that of the ApoE ε3 isoform in *APOE* ε3/ε4 carriers at the tissue level. Because *APOE* expresses most abundantly in astrocytes among these neural cell types, astrocytes might be the primary cell type contributing to this effect. Due to this eQTL effect, the higher proportion of *APOE* ε4 carriers among the patients with AD can lead to a decreased expression observed in the patients, explaining the reported association between the reduced *APOE* expression in astrocytes and the diagnosis of AD [27]. Compared with astrocytes, the *APOE* expression in microglia is upregulated in patients with AD [27]. This overexpression of *APOE* in microglia might be due to either a different regulatory mechanism or a disease-associated subpopulation specifically in microglia, although recent studies have not detected an AD-specific subpopulation in microglia in the cortex of patients with AD [27, 52]. More studies are needed to examine this regulatory effect of the *APOE* ε4 variant at the cell level, including microglia.

Using the single-cell data, we have attempted to pinpoint the cell types that mediate genetic regulatory effects detected at the region-specific analysis. For example, the association between the haplotype containing the GWAS SNP rs2093760 and the ASE of *CR1* observed in the TC was seen in oligodendrocytes but not in microglia. *CR1* is one of the key genes involved in the complement system and shows the highest expression level in oligodendrocytes followed by microglia among the neural cells in our data. Genes in the complement system are abundantly expressed in microglia and are co-expressed with *APOE* [53]. Our findings suggest that oligodendrocytes might play a role in mediating the effect of rs2093760. Nevertheless, this haplotype also includes rs2296160, a non-synonymous variant of *CR1* in almost complete LD with rs2093760. More research is needed to elucidate which functional consequence of this haplotype is the primary factor contributing to the risk of LOAD. Compared to a previous study detecting few *cis*-eQTLs in the primary microglia [43], we identified multiple aseQTLs and eQTLs in microglia, such as *BIN1*, *FCER1G*, *ABCA7*, and *HLA-DQB1*, which were also detected in the region-specific analysis. These findings suggest that microglia might mediate their effects observed at the tissue level.

We have demonstrated using both a simulation study and the real data analysis that the proposed HPMM increases the statistical power by prioritizing the evidence from the allelic imbalance and combining the evidence from both double-heterozygous and homozygous subjects. This method is fast, easy to implement, flexible for adjusting additional covariates and investigating interactions. Our real data analysis also demonstrated that the additional information from the genotype-level expression is important, especially when the evidence from the ASE is limited due to a small sample size. Future work can be focused on further extending the HPMM to accommodate haplotype-based ASE.

It should be noted that the LD between the GWAS SNP and the exonic SNP among the double-heterozygous subjects complicates the interpretation of the findings from the SNP-level aseQTL analysis. It is easier to draw a conclusion when the exonic SNP that is used as the proxy for measuring the ASE is not associated with the ASE intrinsically. Otherwise, we show both theoretically and practically that a false interpretation of the regulatory function can result from an inflated conditional correlation between the exonic proxy SNP and the GWAS SNP among double-heterozygous subjects. We implement a straightforward classification strategy to tackle this issue to gain better biological insights. This problem might not be serious in a haplotype-level allele-specific analysis because the ASE was quantified by aggregating multiple exonic SNPs of the gene.

One of the limitations of the current cell-type allele-specific analysis using the 3’-sequencing snRNA-seq data is that the reads are distributed non-uniformly, and its concentration is biased towards 3’ UTR or 5’ UTR depending on the experimental protocol. Consequently, many exonic SNPs within the gene body are not captured by most reads or covered by a quite low number of reads. This technical limitation leads to low statistical power to detect associations in these regions. Further research using long-read sequencing technologies might offer much better coverage for the aseQTL analysis at the cell level. Another limitation of this study is the small sample size in the snRNA-seq data, which might at least partly justify the lack of significant findings in the cell-type-specific analysis. For loci with low counts or a small sample size, more sophisticated methods based on shrinkage might also be utilized [54].

In conclusion, we detected many AD GWAS loci showing strong associations with the allelic expression of local genes or exons, which can be tissue- or cell-type-dependent. Our results pinpointed the underlying brain regions and cell types that mediate the associations for multiple AD GWAS loci, which provide valuable insights into the cellular regulatory mechanisms in the genetic architecture underlying the pathogenesis of LOAD.

## Methods

### Sample collection

We obtained 747, 572, and 882 bam files of the tissue-level bulk RNA-seq samples in the HCN, PCC, and PFC, respectively, in ROSMAP from Synapse (https://www.synapse.org/#!Synapse:syn22333035). The library was generated using either a poly-A selection or ribosomal depletion method. The reads were trimmed before being aligned to the reference genome (hg19) using Bowtie. These RNA-seq samples were collected from 925 subjects with an age of death ranging from 67.4 to 108.3, among whom 602 are female, and 571 are patients diagnosed with AD by the pathology of the post-mortem brain. We further removed from the downstream analysis five subjects whose genotypes called from the RNA-seq samples were inconsistent with their whole-genome sequencing (WGS) data. The bam files of the 317 bulk RNA-seq samples in the TC from the MayoRNAseq project [26] were downloaded from Synapse (https://www.synapse.org/#!Synapse:syn20818651). This data set included 82 patients with AD, 84 patients with progressive supranuclear palsy (PSP), 71 subjects with pathologic aging, and 78 healthy controls. The reads were aligned to the GRCh38 reference genome using the SNAPR software [55].

We obtained the fastq files of the cell-sorting bulk RNA-seq samples in monocytes and microglia in ROSMAP from Synapse (https://www.synapse.org/#!Synapse:syn11468526 and https://www.synapse.org/#!Synapse:syn22024496). The monocytes were extracted based on the surface marker CD14^+^CD16^-^ from peripheral blood mononuclear cells (PBMCs) of 615 subjects. We included 336 samples that had both the RNA-seq and genotype data, including 238 females and 139 patients with AD. RNA molecules were extracted using the ribosomal depletion method, and the library was prepared using a SMART-seq2 or SMART-seq2-like protocol. The microglia were isolated from the dorsolateral PFC of 10 subjects using magnetic anti CD11b beads and then followed by fluorescence-activated cell sorting based on the CD11b^high^CD45^int^7AAD^-^ staining profile. RNA molecules with a poly-A tail were extracted, and the library was constructed using the SmartSeq-2 protocol [56]. More details about the data generation are given in [40].

We obtained the fastq files of the three snRNA-seq data sets, two in the PFC and one in microglia, in ROMSAP from Synapse (https://www.synapse.org/#!Synapse:syn16780177, https://www.synapse.org/#!Synapse:syn12514624, and https://www.synapse.org/#!Synapse:syn23446265). The two snRNA-seq data sets in the PFC included ~80,000 cells in Brodmann area (BA) 10 from 48 subjects and >160,000 cells from 24 subjects. More details about the generation of these two data sets are described in [27, 38]. In the snRNA-seq data set in microglia, the tissue was extracted from the dorsolateral PFC (BA9/46) of 13 subjects. The microglia were isolated using the same procedure as in the above cell-sorting bulk RNA-seq data. More details about this data set can be found in [39]. All these snRNA-seq libraries were generated using the 10x Genomics Chromium Single Cell 3’ Reagent Kit v2 protocol.

Clinical characteristics of the subjects in ROSMAP and the MayoRNAseq project, including sex, age, race, ethnicity, diagnosis of neurodegenerative disorders, and age at death, were also retrieved from Synapse.

### Quantification of ASE in Bulk RNA-seq data

To process the two bulk RNA-seq data sets in microglia and monocytes that started with fastq files, we first trimmed the paired-end reads using Trim Galore! (https://www.bioinformatics.babraham.ac.uk/projects/trim_galore/). The trimmed fastq files were then aligned to the hg19 human genome assembly using the STAR aligner [57]. Samples with a mapping rate of <50% were excluded from the downstream analysis. Gene-level raw counts were quantitated from the bam file for each sample using the Rsubread package [58] with the parameters “requireBothEndsMapped=TRUE, allowMultiOverlap=TRUE, countMultiMappingReads=FALSE, largestOverlap = TRUE” and the gene transfer format (GTF) build GRCh37.87. In the allele-specific analysis, mapping bias introduced during the library alignment is one source for false positives [59]. We thus used WASP [16] in the STAR aligner to tag and remove reads that showed the alignment bias at exonic SNPs. The exonic SNPs considered in our analysis were defined by those that are included in NCBI dbSNP Build 144 (https://bioconductor.org/packages/release/data/annotation/html/SNPlocs.Hsapiens.dbSNP144.GRCh37.html) and the Haplotype Reference Consortium (HRC) SNP reference panel, and intersect at least one exon defined in the GTF build GRCh37.87. Using the WASP-filtered bam files, the ASE at the exonic SNPs were quantified using the pileup function in the samtools [60] with the parameters “min_base_quality = 10 L, min_mapq=254, distinguish_strands=FALSE, max_depth=10000, include_insertions=TRUE, include_deletions=TRUE” to filter out low-quality and ambiguously mapped reads.

Because the RNA-seq data sets in the PFC, PCC, HCN, and TC are available only in bam files, we re-aligned the reads using WASP to eliminate potential mapping bias. More specifically, we first removed secondary alignments from the original bam files using samtools and re-paired the reads according to the fragment ID using Rsubread. We then used this re-paired bam file as the input in the STAR aligner. The quantification of ASE then followed the same steps described above.

### Quantification of ASE in snRNA-seq data

The raw fastq files from the 10x Genomics platform were processed using STARsolo [61] to obtain the bam files aligned to the hg19 human reference genome. We used WASP to tag and filter those reads that overlapped the exonic SNPs and showed mapping bias. In STARsolo, we used the parameters “--soloCellFilter EmptyDrops_CR --soloCBmatchWLtype 1MM_multi_Nbase_pseudocounts --soloUMIfiltering MultiGeneUMI_CR --soloUMIdedup 1MM_CR” to filter cells and collapse UMI barcodes. We confirmed that this setting generated highly consistent results with CellRanger v3. We first quantified the UMI-level ASE at each of the exonic SNPs for each cell. Specifically, given a cell barcode and an exonic locus, we obtained all UMI barcodes that had at least one read overlapping the locus. Then for each unique UMI barcode, we used the same pileup function as described above to quantify the number of allelic reads that had that UMI barcode and overlapped the locus. We counted only those reads assigned to a transcript by STARsolo. We removed problematic UMIs to which multiple reads were mapped but had different alleles at the exonic locus because this may indicate a sequencing error in one of the reads. Next, we collapsed the UMIs that had at most one mismatch and obtained allele-specific counts by counting the number of collapsed UMIs that shared the same allele. This gave us the UMI-level ASE in each cell at each exonic locus of interest. Finally, we obtained the cell-type-specific UMI-level ASE for each subject by aggregating all cells annotated to the same cell type and subject. We used this ASE in the aseQTL analysis of the snRNA-seq data.

### Genotype data processing, phasing, and imputation

The three genotype data sets in ROSMAP were downloaded from Synapse (https://www.synapse.org/#!Synapse:syn17008936), including a WGS data set of 1196 subjects in the VCF format and two SNP array data sets of 382 (Illumina HumanOmniExpress) and 1709 (Affymetrix GeneChip 6.0) subjects in the plink format. If a subject had both the WGS and SNP array data, we always used the WGS data for better accuracy. Before the imputation, we checked strands, alleles, and positions and removed ambiguous SNPs using the tool HRC-1000G-check-bim.pl with default settings and the reference panel of the EUR population in the 1000 Genomes project (https://www.well.ox.ac.uk/~wrayner/tools/). We phased and imputed the WGS data set using the Michigan Imputation Server [62] with the HRC reference panel (Version r1.1 2016). Because one of the SNP array data sets using the Affymetrix GeneChip 6.0 has many missing data and low-quality genotypes, after removing the subjects and SNPs with a missing rate >5%, 1019 subjects remained. To achieve better imputation quality for the SNP array data, we used the TOPMed imputation reference panel [63] based on hg38 for the phasing and imputation of the SNP array data sets. We mapped the imputed SNPs from hg38 back to hg19 using liftover [64]. The WGS data set of 349 subjects in the MayoRNAseq project was downloaded from Synapse (https://www.synapse.org/#!Synapse:syn10901601). We used the same procedure as in the preparation of the ROSMAP genotype data for the imputation. We imputed the genotype data using the TOPMed imputation reference panel and mapped them back to hg19 using liftover. In the allele-specific analysis, we used only high-quality imputed exonic SNPs that had an imputation quality score>0.96 to control phasing errors.

### Analysis of genotype-level and transcript-level *cis*-eQTLs

The genotype-level *cis*-eQTL analysis using the snRNA-seq data was performed for genes whose TSS was within a ±500k bp window of the AD-associated SNPs. Cell-level count matrices were obtained from the output of STARsolo during the alignment. We included both exonic and intronic reads because pre-mRNA accounts for a large proportion of the snRNA-seq library. We then generated cell-type-specific subject-level pseudo-bulk count matrices by aggregating cells of each subject in each cell type. We adopted the cell type annotation based on the clustering results in [27, 38], which are available from Synapse. The pseudo-bulk raw counts were first normalized using the trimmed mean of M-values (TMM) [65] after removing low-expression genes that had CPM > 1 in <3 subjects. The overdispersion parameters were then estimated using the functions estimateGLMCommonDisp and estimateGLMTagwiseDisp in the edgeR package [66]. Finally, the association tests between the gene expression and the genotypes were performed using the glmFit and glmLRT functions with cohorts as the covariate. In a second model to adjust for the diagnosis of AD, we further added an ordinal variable (between 1 and 5) of the clinical cognitive diagnosis score to the covariates. We also added RNA integrity number (RIN) to the analysis when it is available in a cohort.

In the analysis of transcript-level *cis*-eQTLs using the bulk RNA-seq samples in monocytes, we first generated transcript-level count matrices by quantifying the transcripts from the fastq files using salmon [67] with a pre-built transcriptome index using a partial selective alignment method (http://refgenomes.databio.org/) with the parameters “-l A --validateMappings --rangeFactorizationBins 4”. The downstream analysis followed the same procedure as described above.

### The hierarchical Poisson mixed-effects model for allele-specific eQTL analysis

Testing allelic imbalance is much more powerful than testing genotype-level expression data because the allelic information is generally not subject to technical noises or confounders, which is a severe problem in gene expression analysis. We propose an HPMM that enjoys the power gain from the allelic imbalance and borrows evidence from the expression of homozygous samples to further improve the statistical power. This HPMM is also straightforward to accommodate covariates and interaction terms. The key idea in the HPMM is to introduce two random-effects terms. One accounts for the allele-level overdispersion in the count data, and the other treats the two allelic counts of each subject as paired samples so that the allelic imbalance within the heterozygous samples has higher influence on the inference than the homozygous individual-level expression.

We first describe the HPMM for analyzing the exonic SNPs. The model can be applied with a minor modification for the aseQTL analysis, which will be discussed later. Assume that each heterozygous subject has two allelic counts corresponding to the two alleles *A* and *a*, and only one count is observed for each homozygous subject (*AA* or *aa*). Denote by *y*_*ij*_ the number of RNA-seq reads overlapping the exonic SNP of subject *i*, $i \in \{ 1, \ldots ,n\}$, where *j* represents one of the allele patterns, $j \in \{ A,a,AA,aa\}$. Thus, for a homozygous subject, we have $j = AA\,or\,aa$ depending on the carried allele, while heterozygous subjects have two observations with $j = A\,{{{\mathrm{and}}}}\,a$ (Fig. S4a). Denote by *X*_*ij*_ the coding of the allele patterns defined by

$${{{\boldsymbol{X}}}}_{ij} = \left\{ {\begin{array}{*{20}{c}} {1,\,j = AA\,or\,A} \\ {0,\,j = aa\,or\,a} \end{array}} \right.$$

We use the following HPMM to estimate the exonic aseQTL effect

1 $$y_{ij}\sim Pois({{{\mathrm{exp}}}}(\mu + X_{ij}\beta + {{{\boldsymbol{C}}}}_{ij}\gamma + b_i + \varepsilon _{ij}) \cdot K_{ij}),$$

where *μ* is the intercept, *β* is the effect of the exonic SNP, ${{{\boldsymbol{C}}}}_{ij}$ and ***γ*** are the design matrix and effects of covariates, *b*_*i*_ is the individual-level random effects, *ε*_*ij*_ is the allele-level random effects, both of which follow a zero-mean normal distribution, and $K_{ij}$ is given by

$$K_{ij} = \left\{ {\begin{array}{*{20}{c}} {l_i,\quad {{{\mathrm{if}}}}\,{{{\mathrm{subject}}}}\,{\it{i}}\,{{{\mathrm{is}}}}\,{{{\mathrm{homozygous}}}}} \\ {\frac{{l_i}}{2},\quad {{{\mathrm{if}}}}\,{{{\mathrm{subject}}}}\,{\it{i}}\,{{{\mathrm{is}}}}\,{{{\mathrm{heterozygous}}}}} \end{array}} \right.$$

where *l*_*i*_ is the library size or a normalizing factor. That is, $y_{ij}$ is normalized by half of its total library size if subject *i* is heterozygous. The rationale behind this model is to treat the two observations from a heterozygous subject as paired samples, which is achieved by halving its library size and introducing the random effects *b*_*i*_ to capture the individual-level noises. The HPMM can be easily fitted using, e.g., the glmer function in the lme4 R package [68]. An example R script used in this study for fitting this model can be found in Supplementary Text. If the summary statistics show no sign of overdispersion, *ε*_*ij*_ can be omitted to improve the power. To test whether the allelic imbalance is different between two groups (e.g., the patients with AD and the control group), we add an interaction term to the model

2 $$y_{ij}\sim Pois({{{\mathrm{exp}}}}(\mu + X_{ij}\beta + D_i\alpha + X_{ij}D_i\delta + {{{\boldsymbol{C}}}}\gamma + b_i + \varepsilon _{ij}) \cdot K_{ij}),$$

where *D*_*i*_ ∈ {0, 1} is the diagnosis of the disease. Thus, the differential eQTL effects between case-control groups can be detected by testing *δ* = 0 in model (2).

If there is no homozygous subject, model (1) is equivalent to a binomial model with an overdispersion modeled by *ε*_*ij*_, as shown in [18]. We thus conducted a comprehensive simulation study to compare the statistical power between the proposed HPMM with all subjects, the HPMM with heterozygous subjects only, and the beta-binomial regression [69] implemented by the *betabin* function in the *aod* R package (https://cran.r-project.org/web/packages/aod/index.html). We simulated allelic expression of 500 subjects based on model (1) with *β* ranging from 0 to 0.3, $Var\left( {b_i} \right) = 1$, and $Var\left( {\varepsilon _{ij}} \right) = 0.5$. We considered SNPs with a MAF ranging from 0.1 to 0,4. The empirical power was evaluated at 5% significance level with 2000 simulated replicates. As shown in Fig. S7, all three methods controlled the type I error rate very well (corresponding to effect_size=0). In terms of the statistical power, the beta-binomial regression showed almost the identical performance to the HPMM with heterozygous subjects only. By incorporating homozygous subjects, the proposed HPMM achieved better power than the other two methods. In addition, SNPs with a larger MAF exhibit much larger statistical power because these SNPs have more heterozygous subjects than those with a smaller MAF, suggesting that the number of heterozygous subjects has a large impact.

The aseQTL analysis of AD GWAS SNPs adopted a similar HPMM framework. The difference is that the allelic counts are not directly observed for the GWAS SNPs but are obtained through phased haplotypes as they are not located in an exonic region. Consider haplotypes containing a pair of SNPs, a GWAS SNP with two alleles *A/a* and an exonic SNP with two alleles *B/b*, where *a* and *b* are the minor alleles. Denote by $y_{ij}$ the number of reads observed for subject *i* carrying the allele pattern *j* of GWAS SNP, $j \in \{ A,a,AA,aa,Aa\}$. We split those subjects being heterozygous at both GWAS and exonic loci (i.e., double-heterozygous) into two observations (that is, for subject *i* who is double-heterozygous, we have $j = \{ A,a\}$) (Fig. S4b) because allelic reads can be determined for these subjects. For each double-heterozygous subject, we have two observations corresponding to the haplotypes on the two chromosomes (i.e., either (1) $j = A,\,k = B$ and $j = a,\,k = b$, or (2) $j = A,\,k = b$ and $j = a,\,k = B$), where $k \in \{ B,b\}$ is the allele of the exonic SNP. The library size of each allelic observation is one-half of the library size of the subject. Subjects who are not double-heterozygous have only one observation (i.e., $j = AA\,{{{\mathrm{or}}}}\,aa\,{{{\mathrm{or}}}}\,Aa$). Note that $j = Aa$ is used for those who are heterozygous at the GWAS SNP but not at the exonic SNP. Denote by *X*_*ij*_ the variable coded for the GWAS SNP defined by

$${{{\boldsymbol{X}}}}_{ij} = \left\{ {\begin{array}{*{20}{l}} {1,\,j = AA\,or\,A} \hfill \\ {0.5,\,j = Aa} \hfill \\ {0,\,j = aa\,or\,a} \hfill \end{array}} \right..$$

In this study, we fitted model (1) using this definition of *X*_*ij*_, and the estimated *β* was the aseQTL effect of the AD GWAS SNP (See Supplementary Text for an example script).

The interpretation of the allelic analysis of a GWAS SNP is more complicated than that of an exonic aseQTL because the allelic reads are not directly measured for the GWAS SNP. Like GWAS, the LD between the two SNPs complicates the interpretation. Because the double-heterozygous subjects are treated like paired samples in model (1), they have a large impact on the estimate of *β*. Therefore, the correlation between alleles in the double-heterozygous subjects is more influential. It should be noted that this allelic correlation between the GWAS and exonic SNPs in double-heterozygous subjects can be very large even if the two SNPs are not in LD at the population level, particularly when the sample size is small or the MAF is low (Fig. S5). For example, following the previous notations, we consider a situation where there is no LD between the two SNPs. In this case, the haplotype relative frequency equals the product of allele frequency, that is, *P*_*ab*_ = *P*_*a*_*P*_*b*_. If the MAF is small (i.e., *P*_*a*_ or *P*_*b*_ is small), then *P*_*ab*_ is nearly zero. Thus, under a small sample size, we may not even observe subjects with the haplotype *ab* in double-heterozygous subjects. The lack of this haplotype means that the only possible combination in the double-heterozygous subjects is $j = A,\,k = b$ and $j = a,\,k = B$, which leads to complete allelic correlation.

### Quality control in the allele-specific analysis

We performed stringent quality control for sequencing error and mapping bias, which might significantly affect the allele-specific analysis as mentioned in [70]. As described above, WASP was used in all the analyses, and only those reads with tag vW=1 were retained. In the pileup function, we counted only high-quality reads that were mapped uniquely and had a minimum base Quality=10 and minimum MAPQ=254. To minimize potential sequencing and phasing errors, we chose to use the imputed genotypes for the exonic SNPs, but we also double-checked them with the genotypes called from the RNA-seq data. We adopted genotypes called from the RNA-seq data if the two genotypes were inconsistent. The criterion for the inconsistency was defined by either the ratio between the counts of two alleles >10% when the imputed genotype was homozygous or the ratio <2% when the imputed genotype was heterozygous if >20 reads overlapped the locus in the RNA-seq data. As the correct phase information is critical for the accuracy of the allele-specific analysis, we examined only those SNPs with an imputation quality score *R*^2^ > 0.96.

### Functional annotation and external resource

To compare the eQTL results in CD14+CD16- classical monocytes, we obtained the VCF files containing the summary statistics of significant eQTLs in classical monocytes identified in the DICE. To compare the eQTLs in the brain regions, we obtained the summary statistics of significant eQTLs from GTEx (v8) in three brain tissues, including the PFC, ACC, and hippocampus [13], and from a meta-analysis of the TC and PFC conducted in [12]. The summary statistics of sQTLs in the PFC were obtained from a supplementary table of [34]. The summary statistics of gene-level, transcript-level, exon-level eQTLs in BrainSeq [33] were downloaded from http://eqtl.brainseq.org/phase1/eqtl/. The summary statistics of gene-level and transcript-level eQTLs in CommonMind [32] were downloaded from the FTP server of the eQTL Catelogue (https://www.ebi.ac.uk/eqtl/). The functional annotation of the exonic variants in the aseQTL analysis was performed using the VariantAnnotation R package [71], in which we annotated the location of the exonic variants using the database *TxDb.Hsapiens.UCSC.hg38.knownGene*, the amino acid coding change (non-synonymous, synonymous, frameshift, and nonsense), and prediction of the impact of non-synonymous variants using PolyPhen (Polymorphism Phenotyping).

## Supplementary information

Supplementary legends

Table S1

Table S2

Table S3

Table S4

Table S5

Table S6

Table S7

Table S8

Table S9

Figure S1

Figure S2

Figure S3

Figure S4

Figure S5

Figure S6

Figure S7

Supplementary Text

## Data Availability

An example R script for implementing the HPMM used in this study is provided in Supplementary Text.
