# Supplementary material for: Allele-specific analysis reveals exon- and cell-type-specific regulatory effects of Alzheimer’s disease-associated genetic variants
Source: Transl Psychiatry. 2022 Apr 18;12:163. doi: 10.1038/s41398-022-01913-1 (PMC9016079; doi:10.1038/s41398-022-01913-1)
Supplement: Supplementary file 1 — Supplementary legends [file 41398_2022_1913_MOESM1_ESM.docx]

# **Supplementary Tables**

Table S1. Summary statistics of the analysis of aseQTLs for the AD-associated SNPs in the four brain regions using the bulk RNA-seq data. AD SNP: the rs ID of the AD-associated SNP. AD SNP Info: the detailed information of the AD-associated SNP including chromosome, genomic location (hg19), reference allele, and effect allele. Exonic SNP Info: the detailed information of the exonic variant used for measuring the ASE. Mean expression count: the mean raw count of the expression measured at the exonic variant. Beta (P) of AD SNP: the logFC (p-value) of the effect of the AD-associated SNP on the expression. Beta (P) of exonic SNP: the logFC (p-value) of the effect of the exonic variant on the expression. Sample size: Actual sample size in testing this association. Gene name: the gene where the exonic SNP is located. Amino acid modification, Exonic region, Prediction for nonsynonymous: Functional annotation of the exonic SNP. Padj: FDR adjusted p-value. Category: Classification of significant aseQTLs.

Table S2. Summary statistics of the interaction analysis between the age group and the genotype for the significant AD-associated SNPs detected in the four brain regions. Exonic SNP pos: the genomic position (hg19) of the exonic variant used for measuring the ASE. Mean count: the mean raw count of the expression measured at the exonic variant. P of interaction: the p-value of the interaction term between the age group and the genotype. Adjust P of interaction: FDR adjusted p-value.

Table S3. Summary statistics of the analysis of aseQTLs for the AD-associated SNPs in the six neural cell types using the snRNA-seq data. AD SNP: the rs ID of the AD-associated SNP. AD SNP Info: the detailed information of the AD-associated SNP including chromosome, genomic location (hg19), reference allele, and effect allele. Exonic SNP Info: the detailed information of the exonic variant used for measuring the ASE. Mean expression count: the mean raw count of the expression measured at the exonic variant. Beta (P) of AD SNP: the logFC (p-value) of the effect of the AD-associated SNP on the expression. Beta (P) of exonic SNP: the logFC (p-value) of the effect of the exonic variant on the expression. Gene name: the gene where the exonic SNP is located. Amino acid modification, Exonic region, Prediction for nonsynonymous: Functional annotation of the exonic SNP. Padj: FDR adjusted p-value.

Table S4. Summary statistics of the analysis of genotype-level *cis*-eQTLs for the AD-associated SNPs in the six neural cell types using pseudo-bulk samples by aggregating cells in the snRNA-seq data. logCPM: logarithm of the count per million of the gene expression. Adjusted P: FDR adjusted p-value. SNP: the SNP ID, the effect allele (first), and the reference allele (second).

Table S5. Summary statistics of the analysis of aseQTLs for the AD-associated SNPs in microglia using the combined snRNA-seq and cell-sorting bulk RNA-seq data. AD SNP: the rs ID of the AD-associated SNP. AD SNP Info: the detailed information of the AD-associated SNP including chromosome, genomic location (hg19), reference allele, and effect allele. Exonic SNP Info: the detailed information of the exonic variant used for measuring the ASE. Mean expression count: the mean raw count of the expression measured at the exonic variant. Beta (P) of AD SNP: the logFC (p-value) of the effect of the AD-associated SNP on the expression. Beta (P) of exonic SNP: the logFC (p-value) of the effect of the exonic variant on the expression. Sample size: Actual sample size in testing this association. Gene name: the gene where the exonic SNP is located. Amino acid modification, Exonic region, Prediction for nonsynonymous: Functional annotation of the exonic SNP. Padj: FDR adjusted p-value.

Table S6. Summary statistics of the analyses of genotype-level *cis*-eQTLs for the AD-associated SNPs in microglia using pseudo-bulk samples by aggregating cells in the combined snRNA-seq and cell-sorting bulk RNA-seq data. The first analysis is adjusted for batch, and the second analysis is adjusted for both batch and the diagnosis of AD. logCPM: logarithm of the count per million of the gene expression. Adjusted P: FDR adjusted p-value. SNP: the SNP ID, the effect allele (first), and the reference allele (second).

Table S7. Summary statistics of the analysis of aseQTLs for the AD-associated SNPs in monocytes using the cell-sorting bulk RNA-seq data. AD SNP: the rs ID of the AD-associated SNP. AD SNP Info: the detailed information of the AD-associated SNP including chromosome, genomic location (hg19), reference allele, and effect allele. Exonic SNP Info: the detailed information of the exonic variant used for measuring the ASE. Mean expression count: the mean raw count of the expression measured at the exonic variant. Beta (P) of AD SNP: the logFC (p-value) of the effect of the AD-associated SNP on the expression. Beta (P) of exonic SNP: the logFC (p-value) of the effect of the exonic variant on the expression. Sample size: Actual sample size in testing this association. Gene name: the gene where the exonic SNP is located. Amino acid modification, Exonic region, Prediction for nonsynonymous: Functional annotation of the exonic SNP. Padj: FDR adjusted p-value.

Table S8. Summary statistics of the eQTL analysis of rs3865444 with the transcript-level expression of *CD33*.

Table S9. A list of the source of each of the 31 SNPs (excluding the well-known *APOE* ε2 and *APOE* ε4 variants) under investigation.

# **Supplementary Figures**

Figure S1. Allelic and genotype-level expression plots of the eleven significant associations that are identified in the aseQTL analysis in monocytes and are not shown in Fig. 6. The boxplots summarize the genotype-level expression, and the ASE of the double-heterozygous samples (yellow points) are shown in the scatter plot below. The dashed line is a smooth curve fitted using linear regression.

Figure S2. Three significant associations identified in the aseQTL analysis in microglia between (a) rs59735493 and *ITGAM*, (b) rs2081545 and *MS4A7,* and (c) rs12590654 and *ATXN3*. ASE_NA: those subjects with unknown ASE because they are not double-heterozygous. The pair of the ASE of a double-heterozygous subject are connected by a dashed line. The CPM of the ASE of the double-heterozygous subjects is normalized by half of its total library size.

Figure S3. Significant associations between (a) rs1859788 and the ASE of *TRIM4*, and (b) rs3740688 and the ASE of *MTCH2* in the excitatory neurons. The boxplots summarize the genotype-level expression. The ASE of the double-heterozygous subjects (yellow points) are shown in the scatter plot. The dashed line is a smooth curve fitted using linear regression.

Figure S4. The coding strategy used in the proposed HPMM for the aseQTL analysis of the association (a) between an exonic SNP and its ASE and (b) between a GWAS SNP and the ASE measured at an exonic SNP.

Figure S5. The difference between the genotype correlation among the whole sample and the allelic correlation among double-heterozygous subjects observed in the 367 significant associations (FDR p<0.05) identified in the analysis of aseQTLs in the four brain regions. X-axis: the product of the MAF of the AD-associated SNP and the exonic SNP. The color of the points indicates whether the genotype correlation among the whole sample is positive or negative.

Figure S6. The distribution of the subjects in ROSMAP that had bulk RNA-seq samples in multiple brain regions. Those subjects whose genotypes are inconsistent between the RNA-seq samples and their WGS data are not included in these numbers. The plot shows that 472 subjects had an RNA-seq sample in each of the three brain regions.

Figure S7. Comparison of the empirical statistical power between the HPMM using both heterozygous and homozygous samples, the HPMM using the heterozygous samples only, and the beta-binomial model.

# **Supplementary Text**

An example R script for implementing the HPMM used in this analysis along with an example dataset.
