# Supplementary figures and images for: Allele-specific analysis reveals exon- and cell-type-specific regulatory effects of Alzheimer’s disease-associated genetic variants
Source: Transl Psychiatry. 2022 Apr 18;12:163. doi: 10.1038/s41398-022-01913-1 (PMC9016079; doi:10.1038/s41398-022-01913-1)

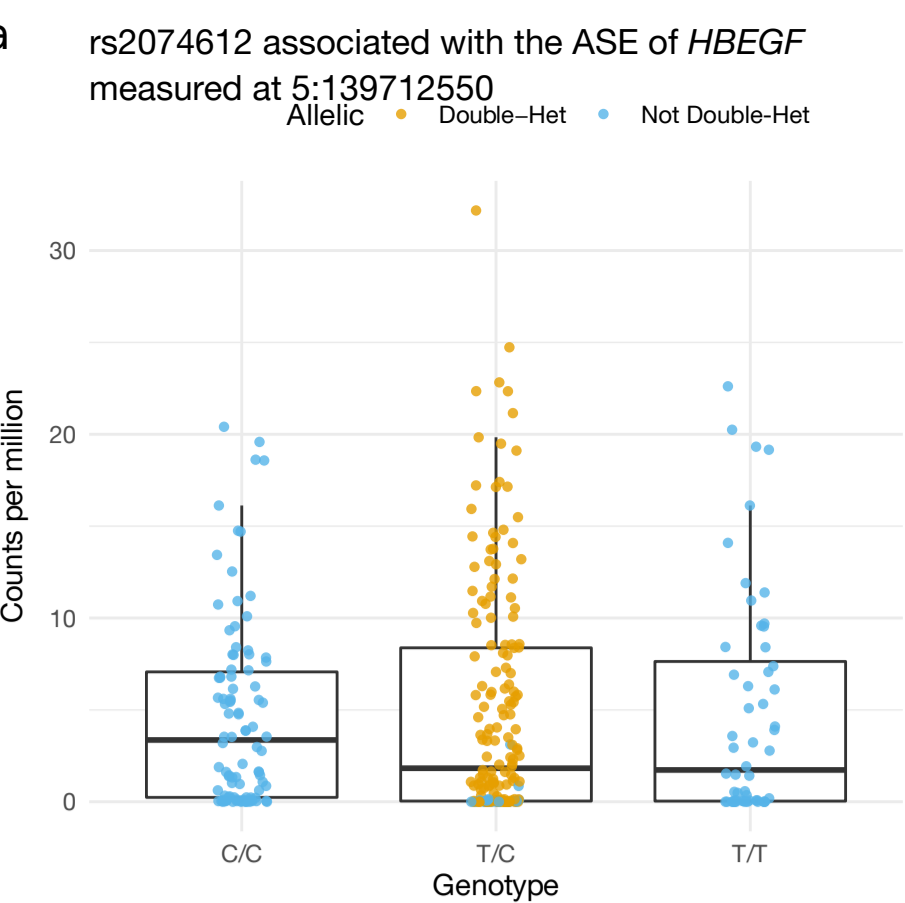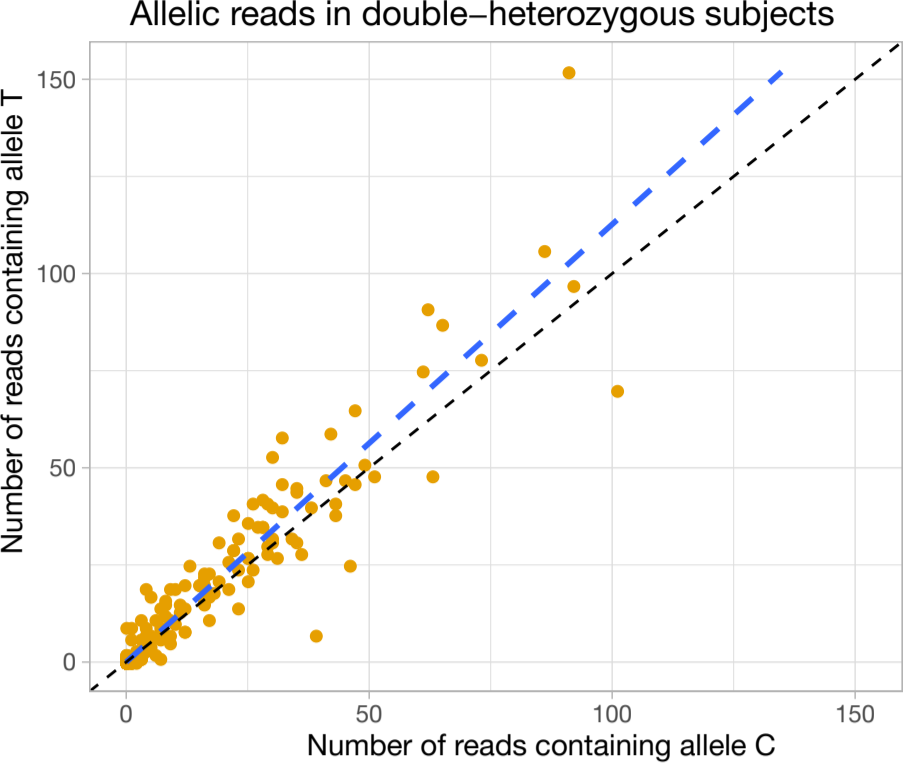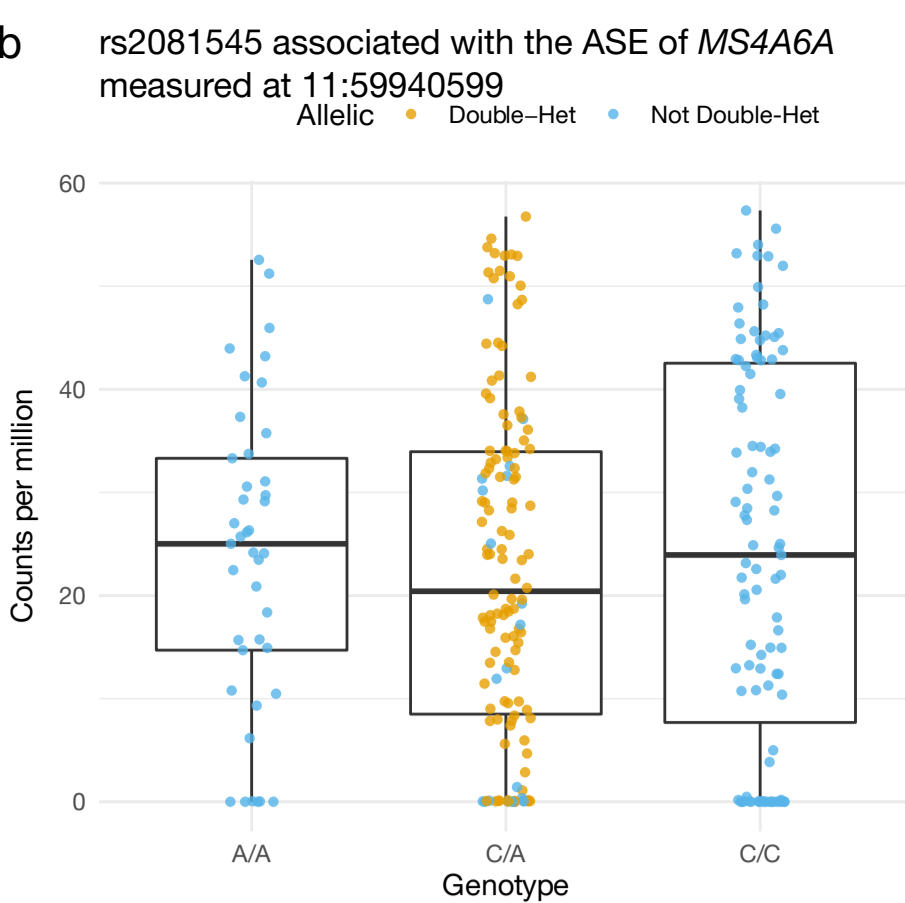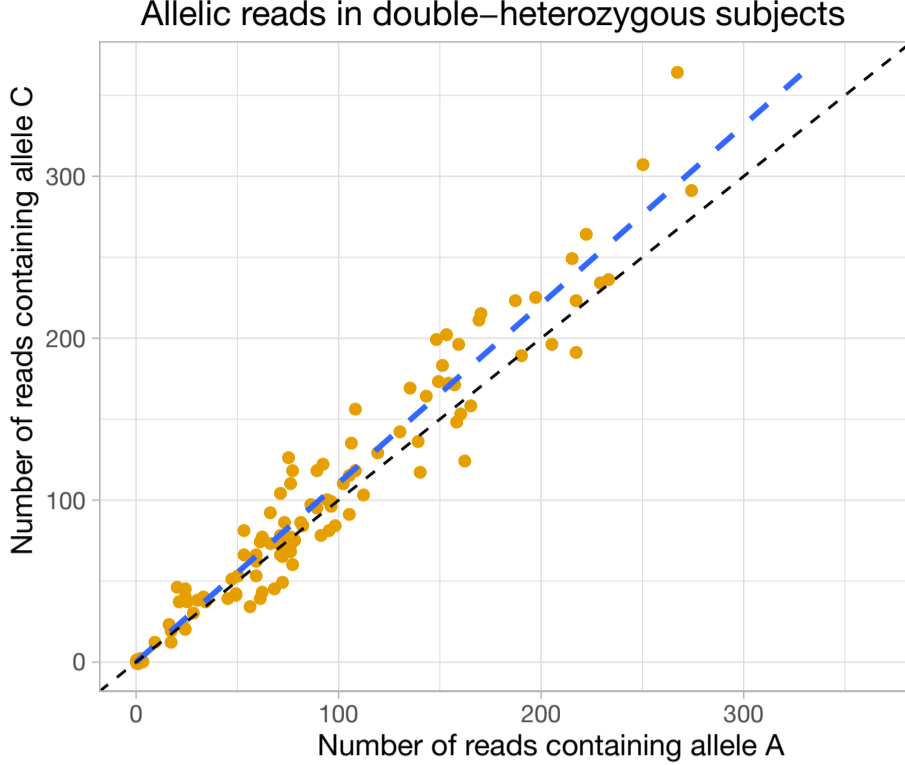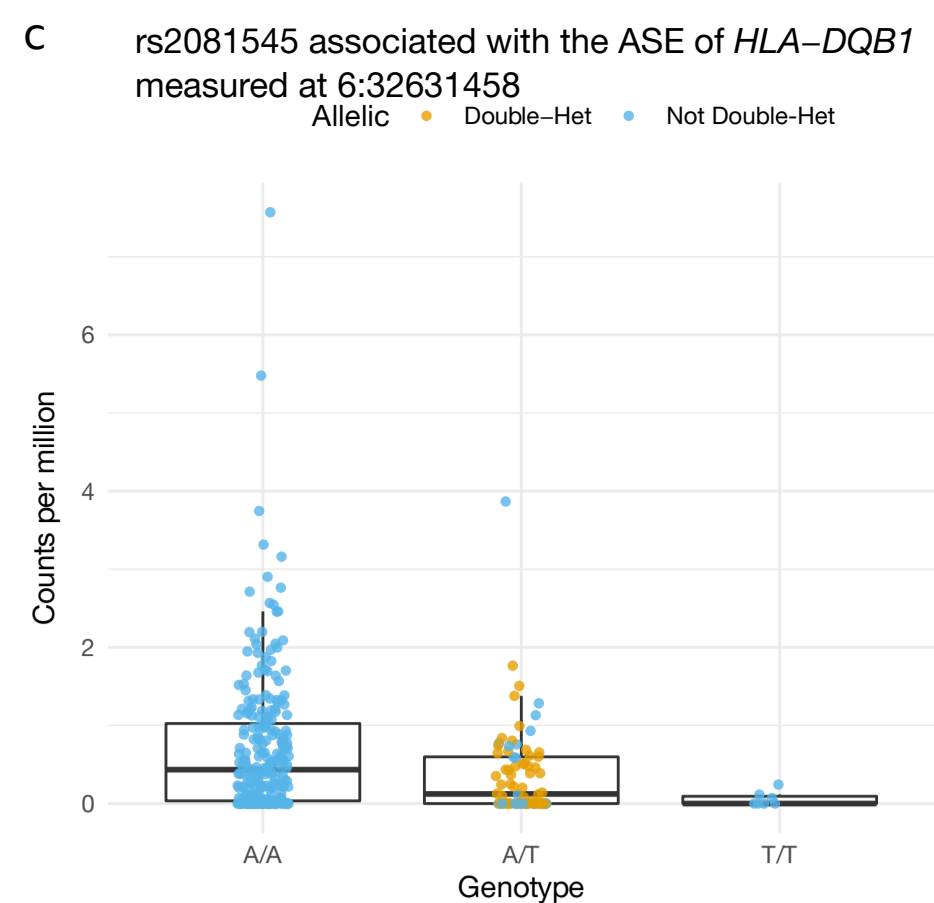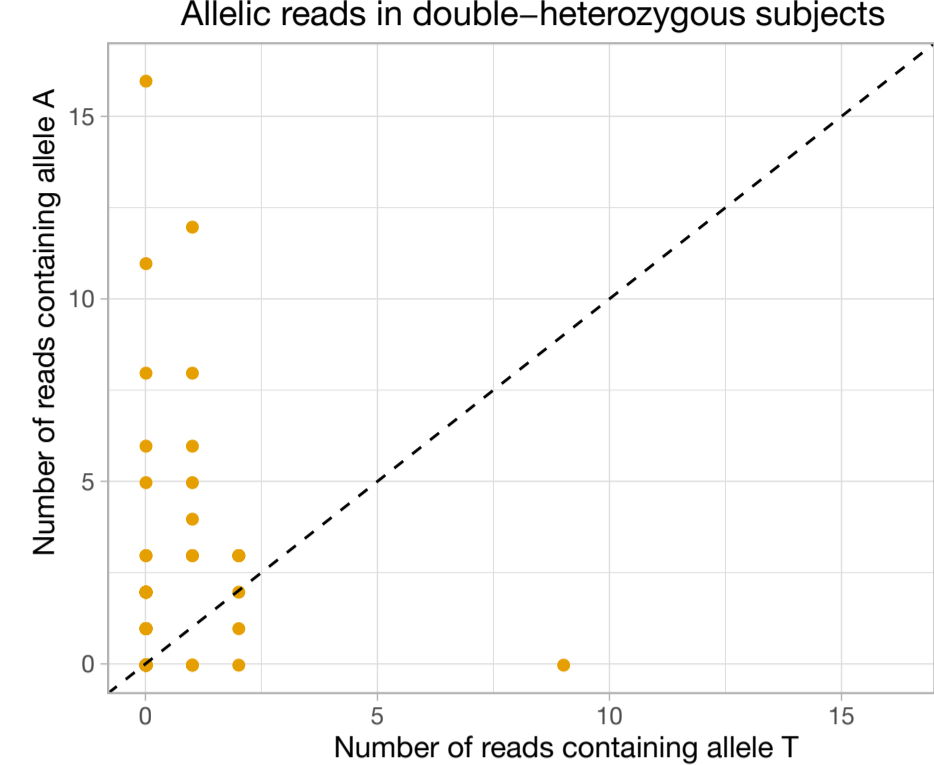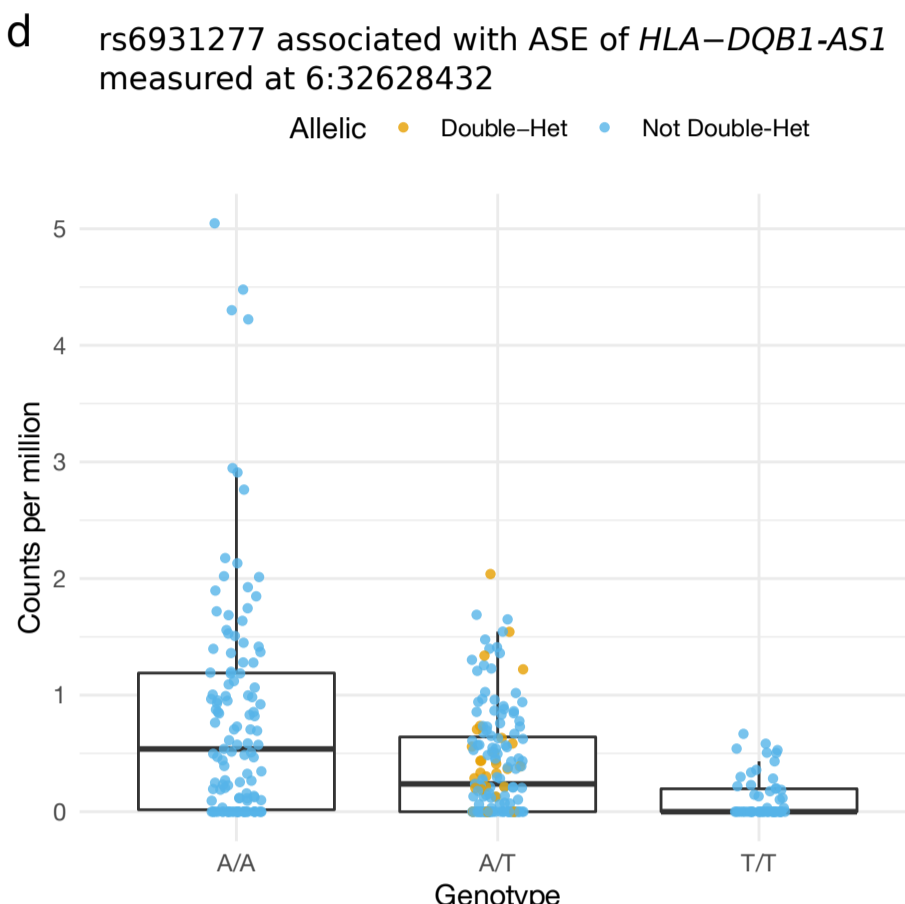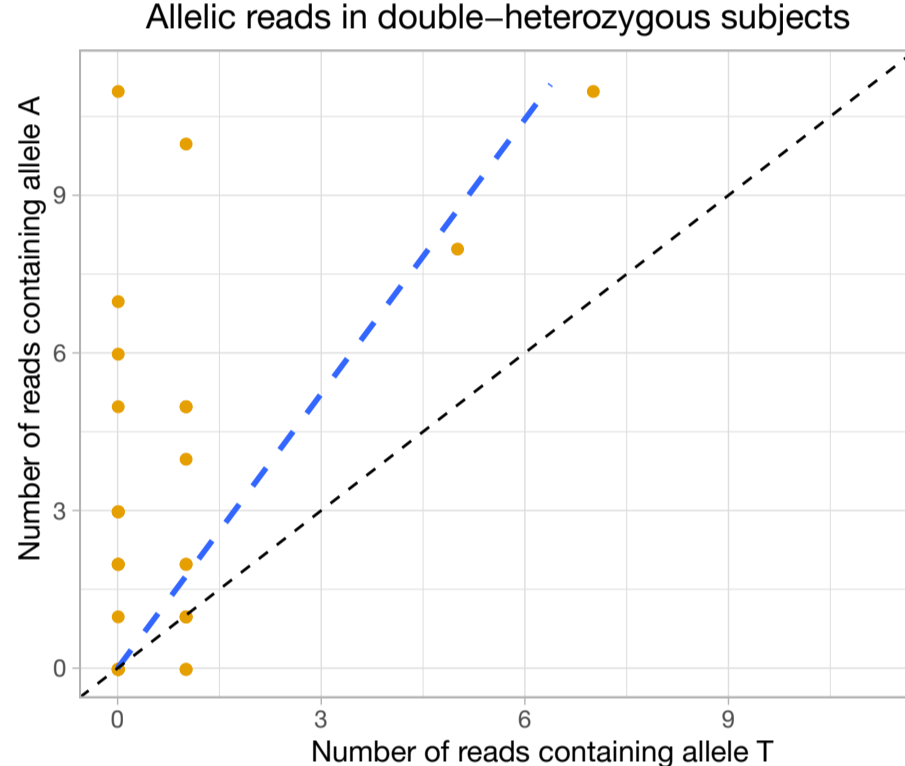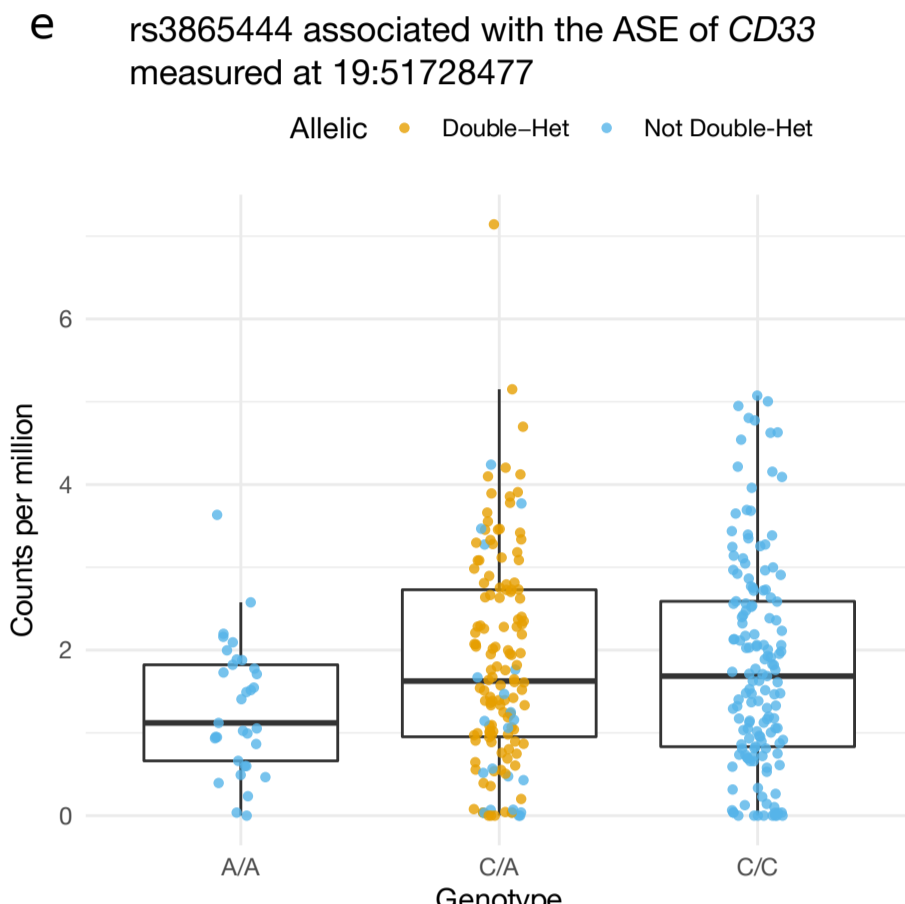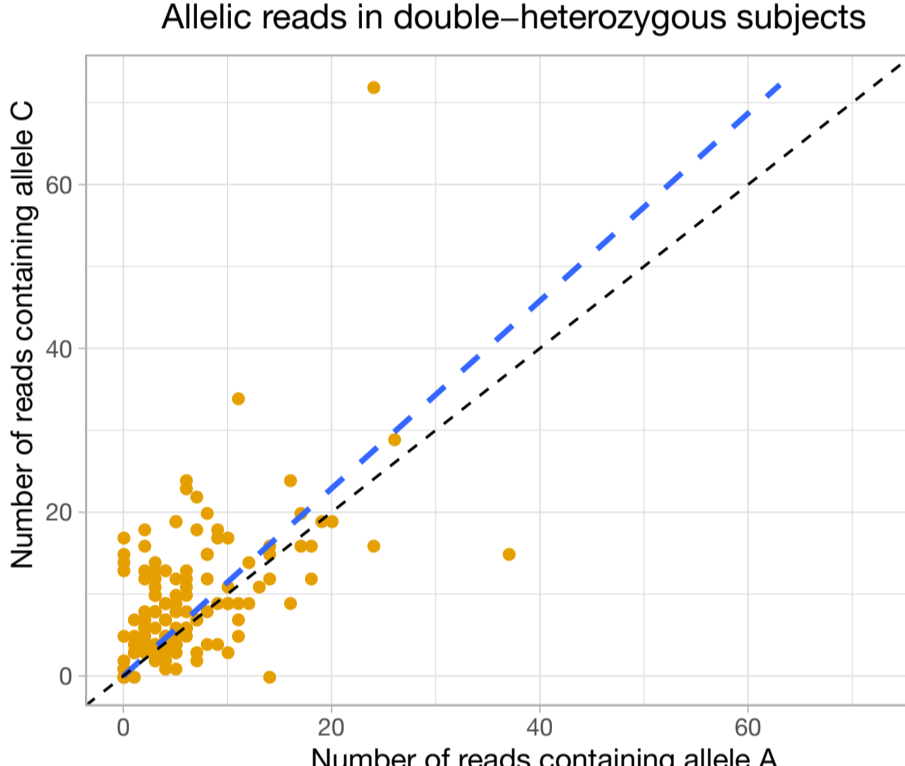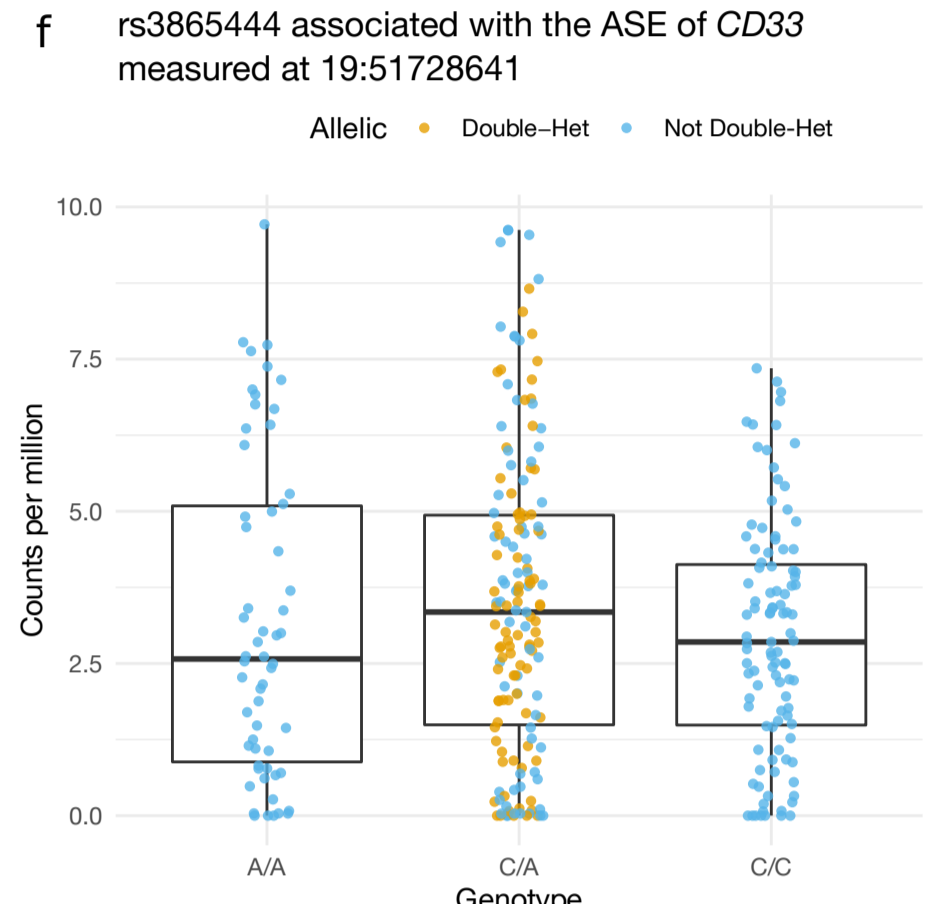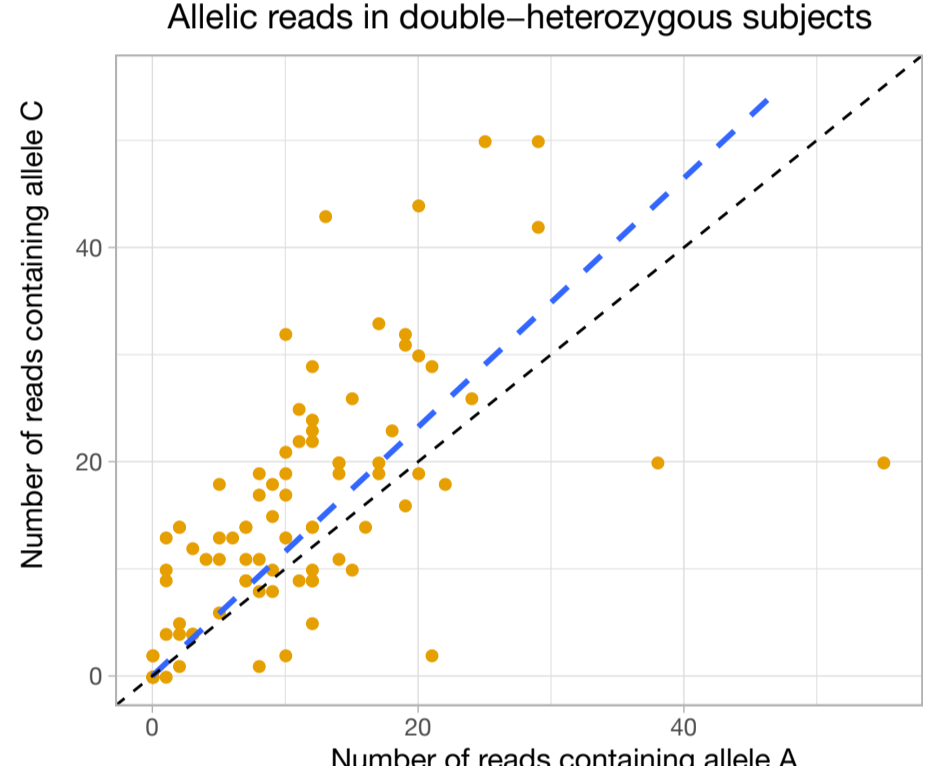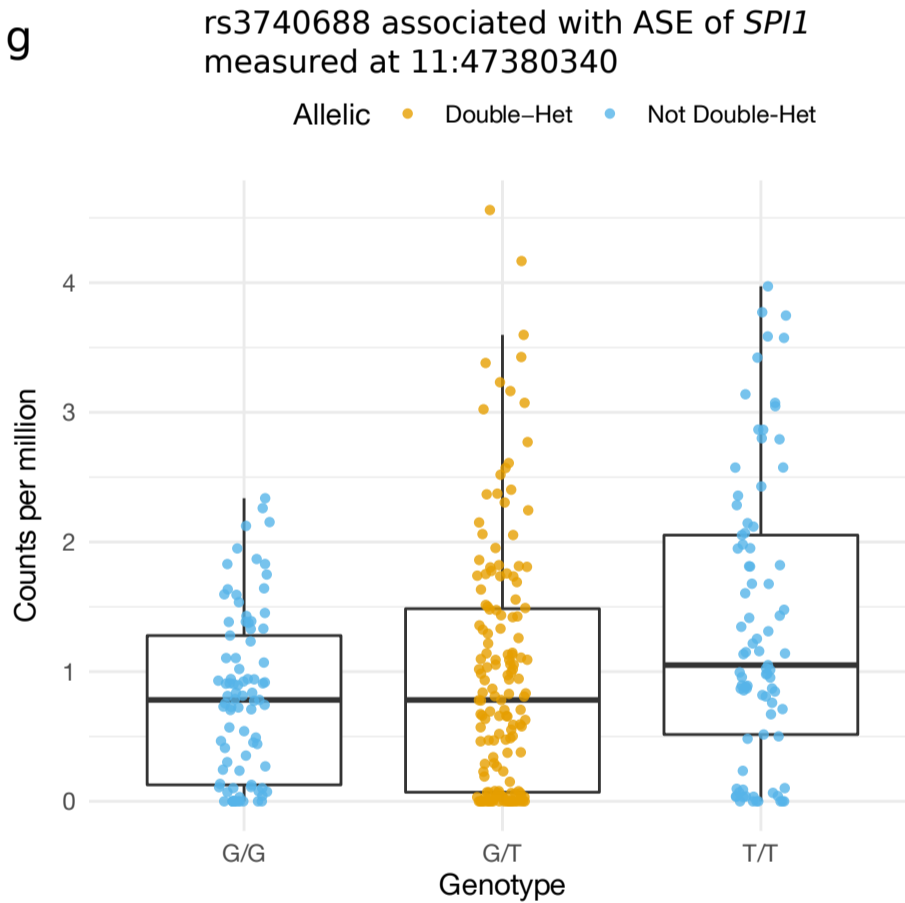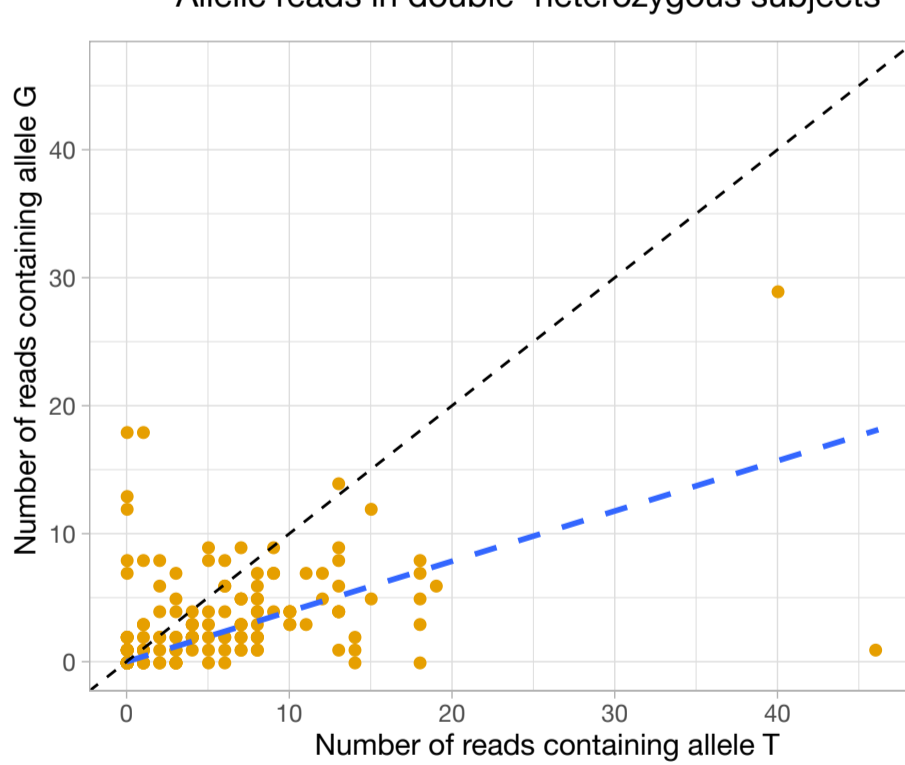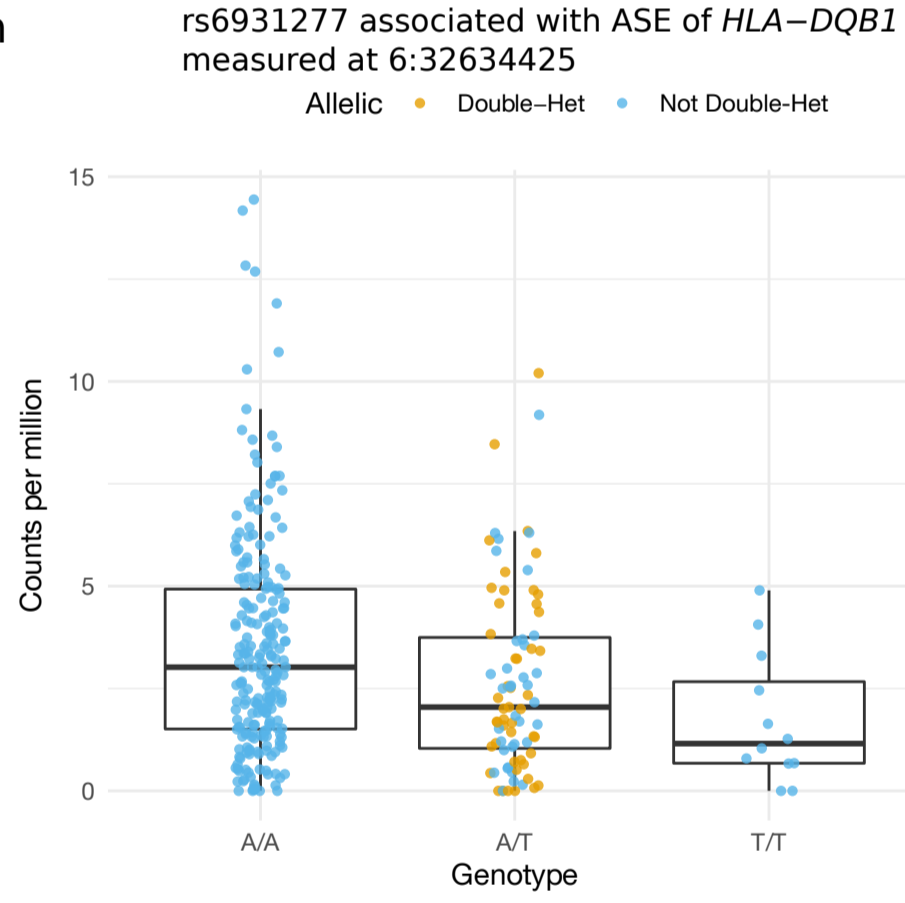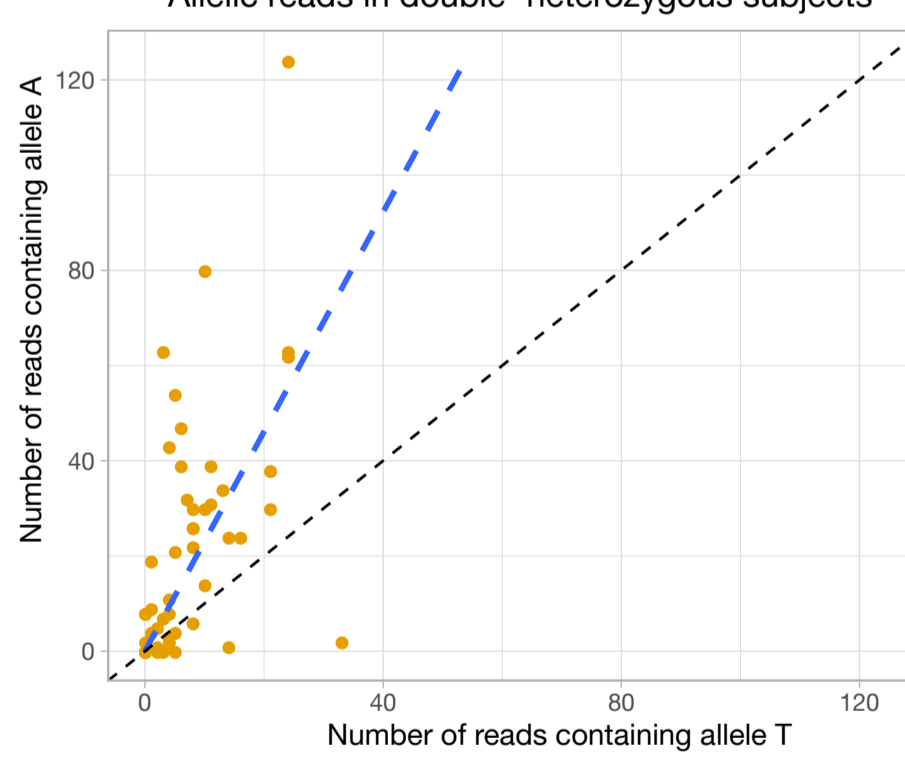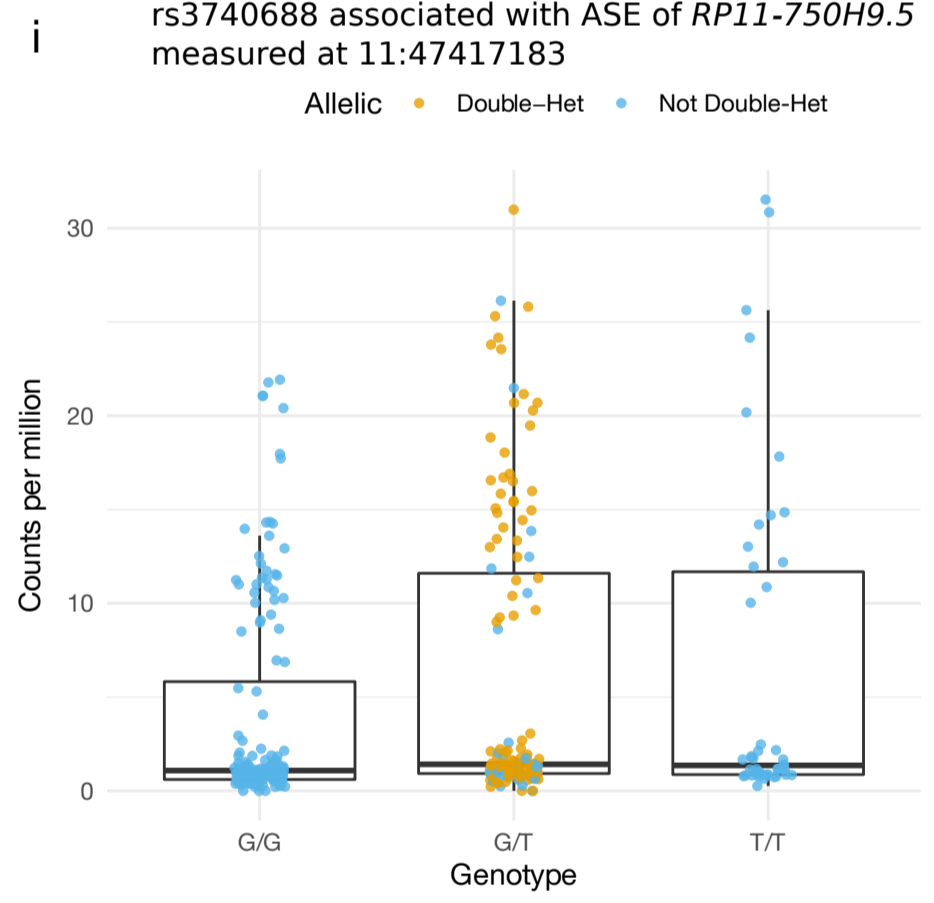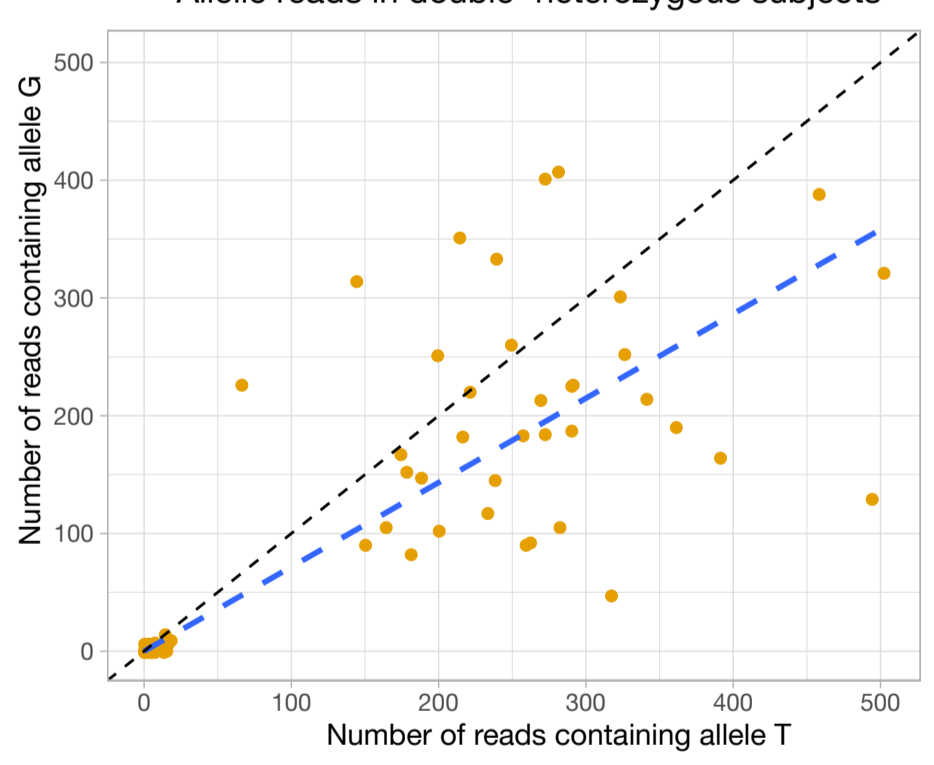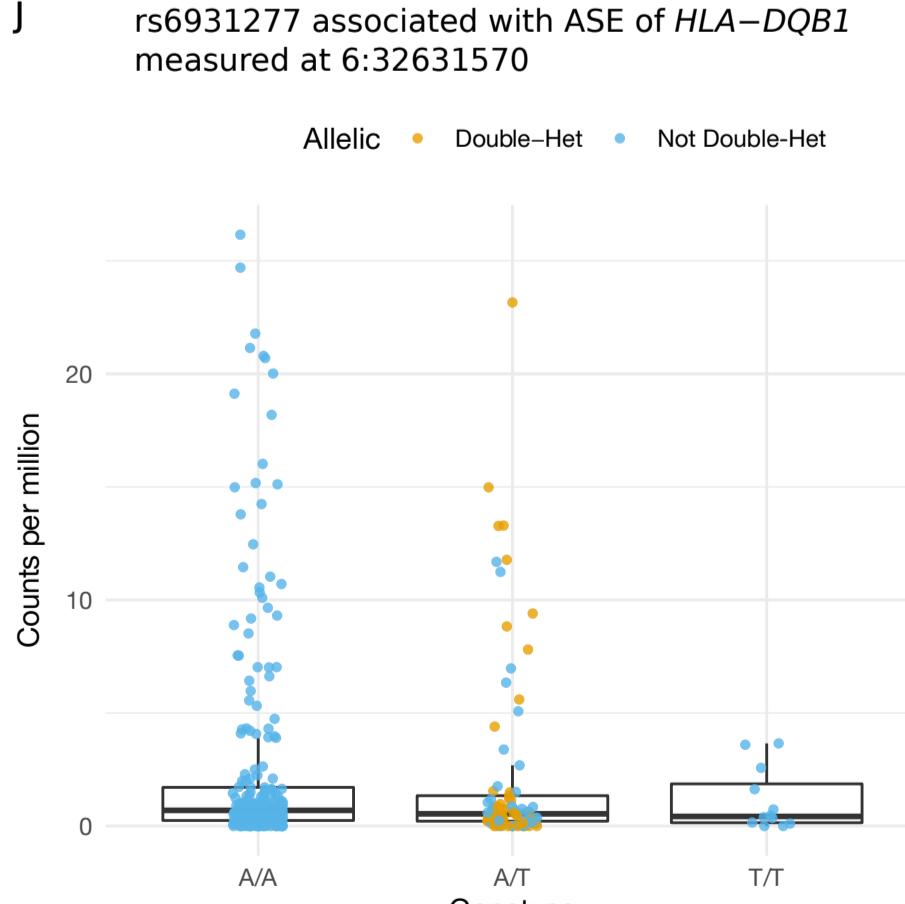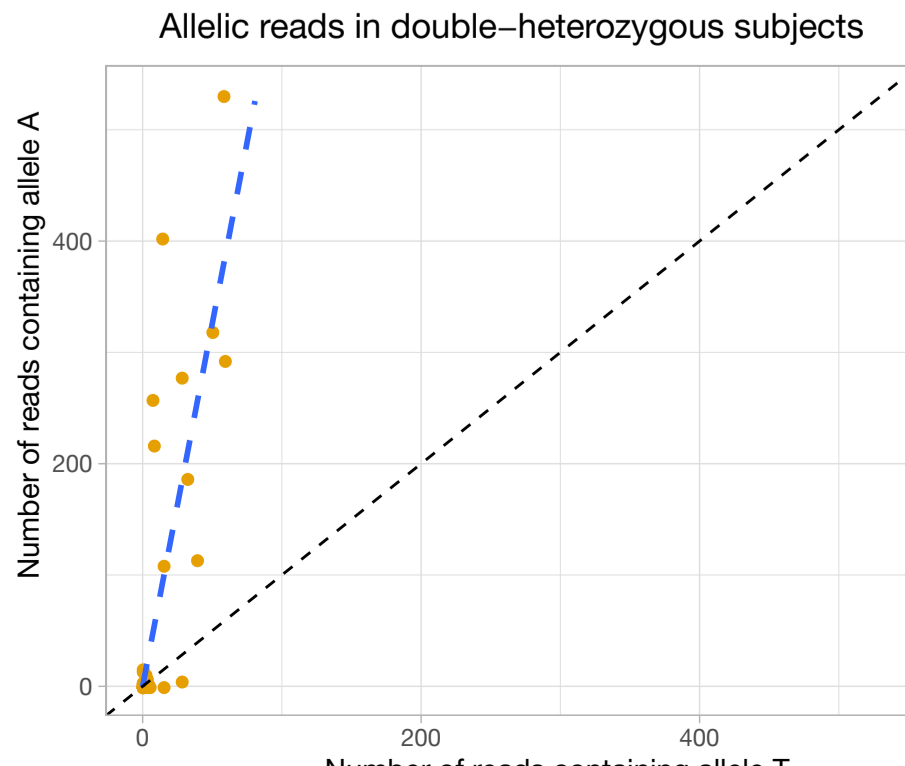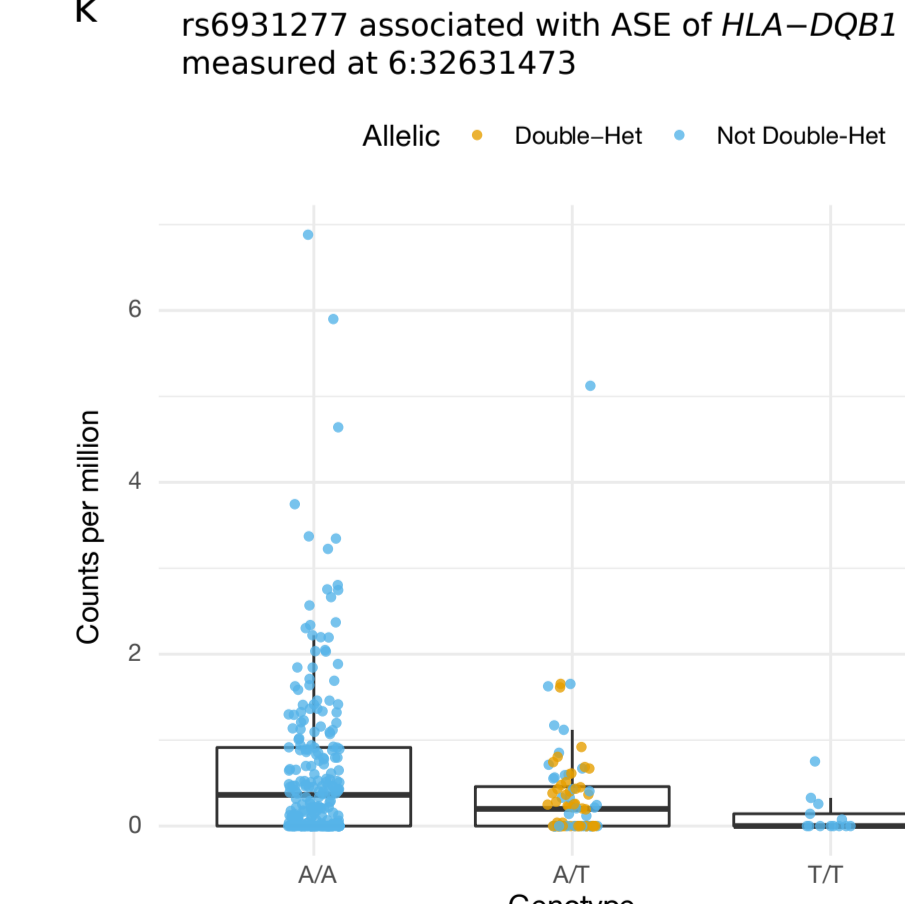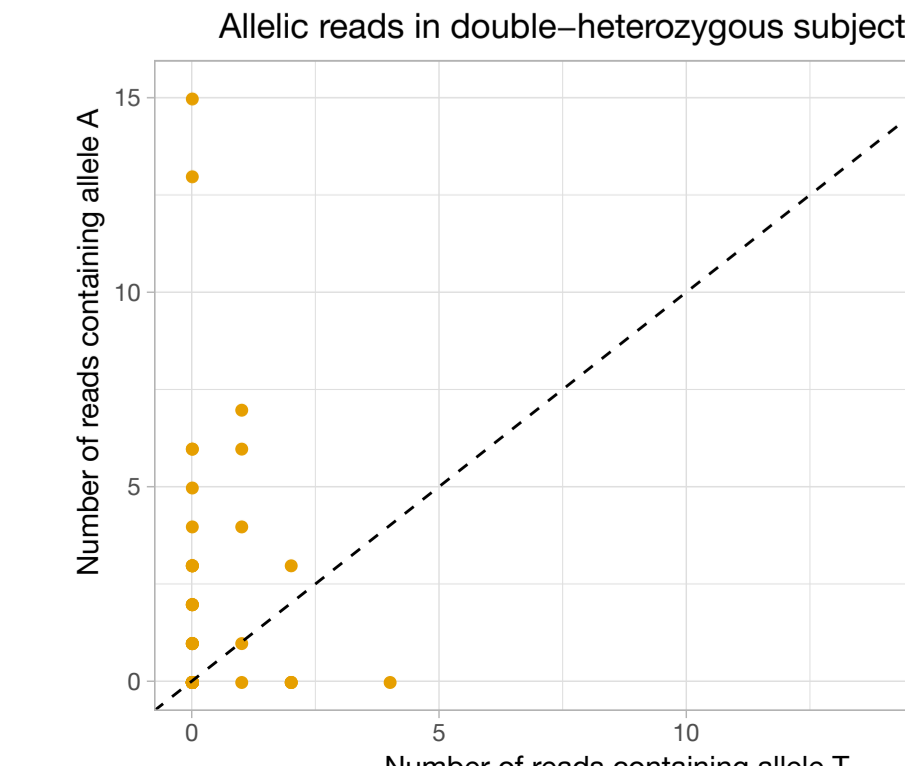

Supplement: Supplementary file 11 — Figure S1 [file 41398_2022_1913_MOESM11_ESM.pdf]

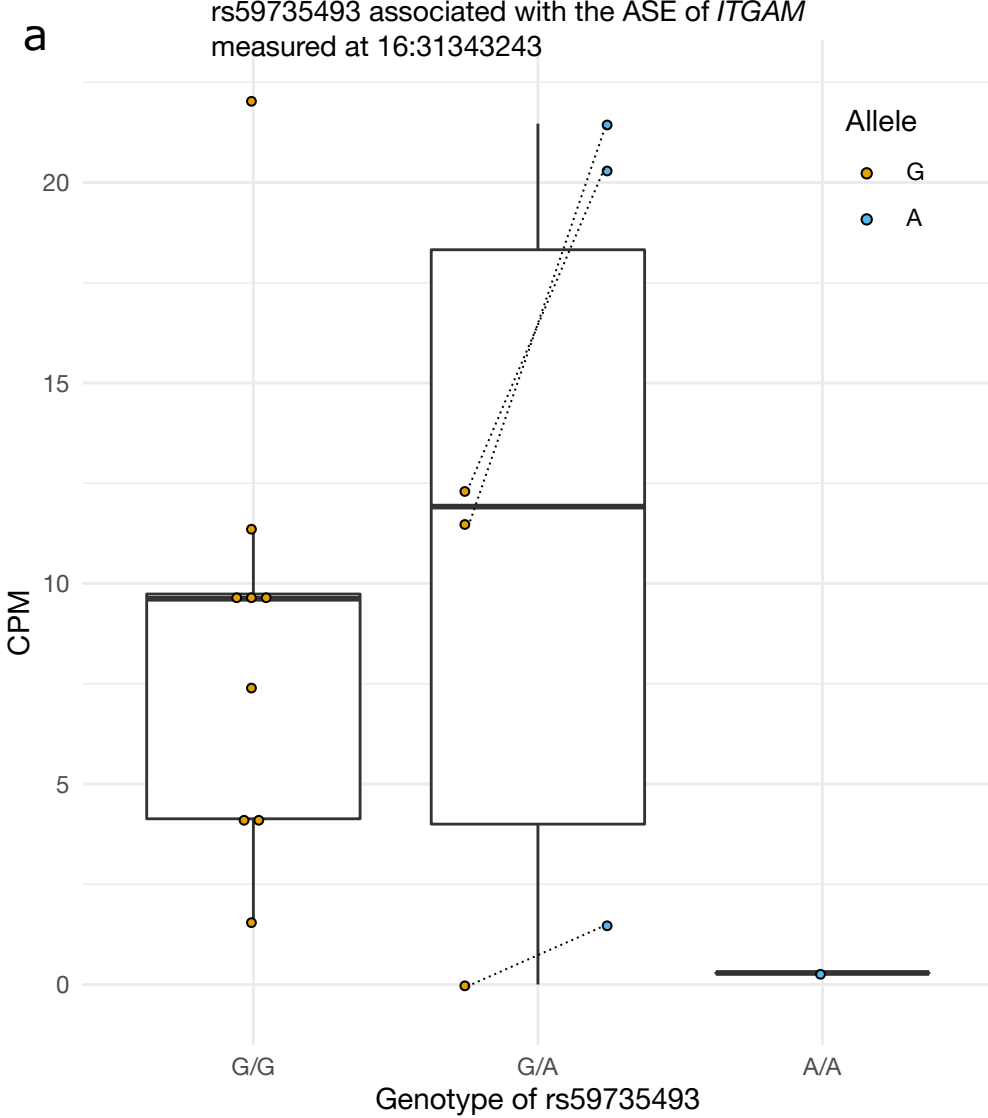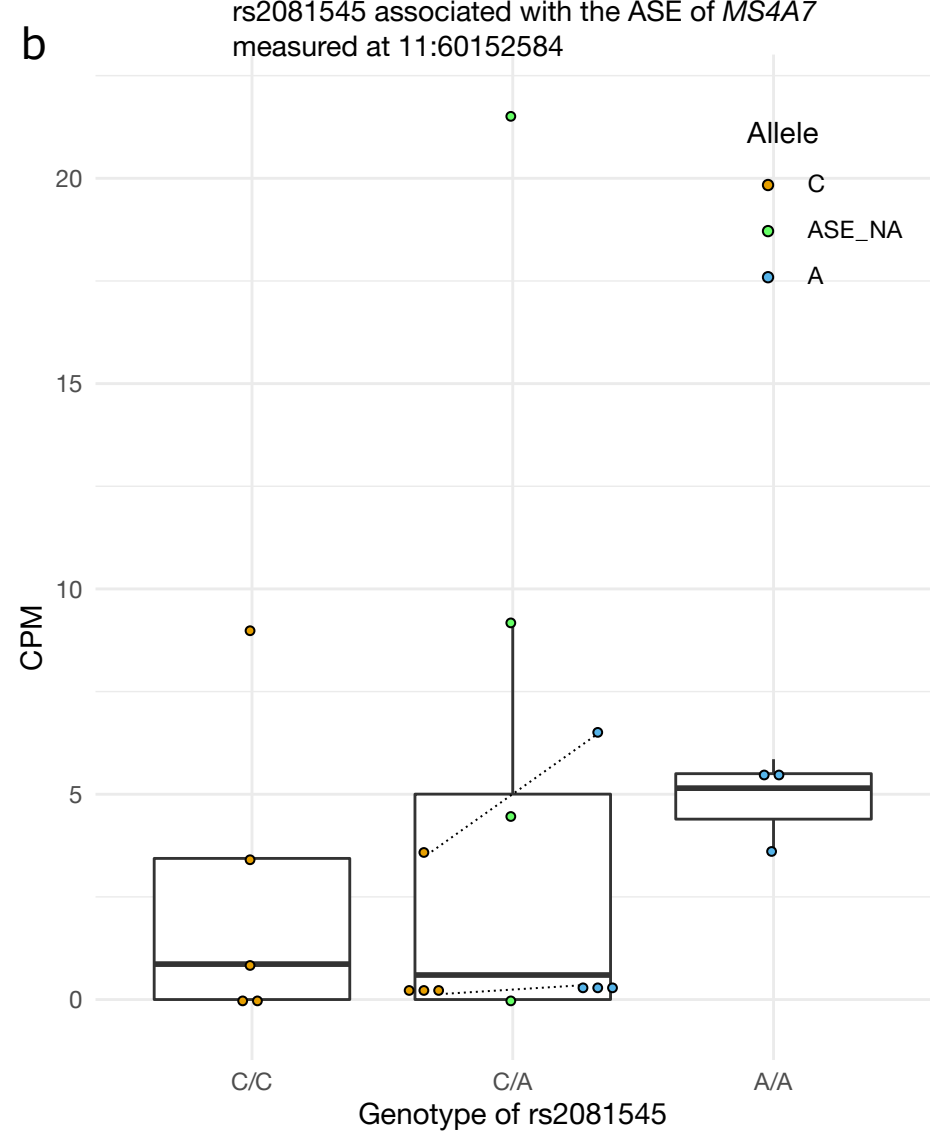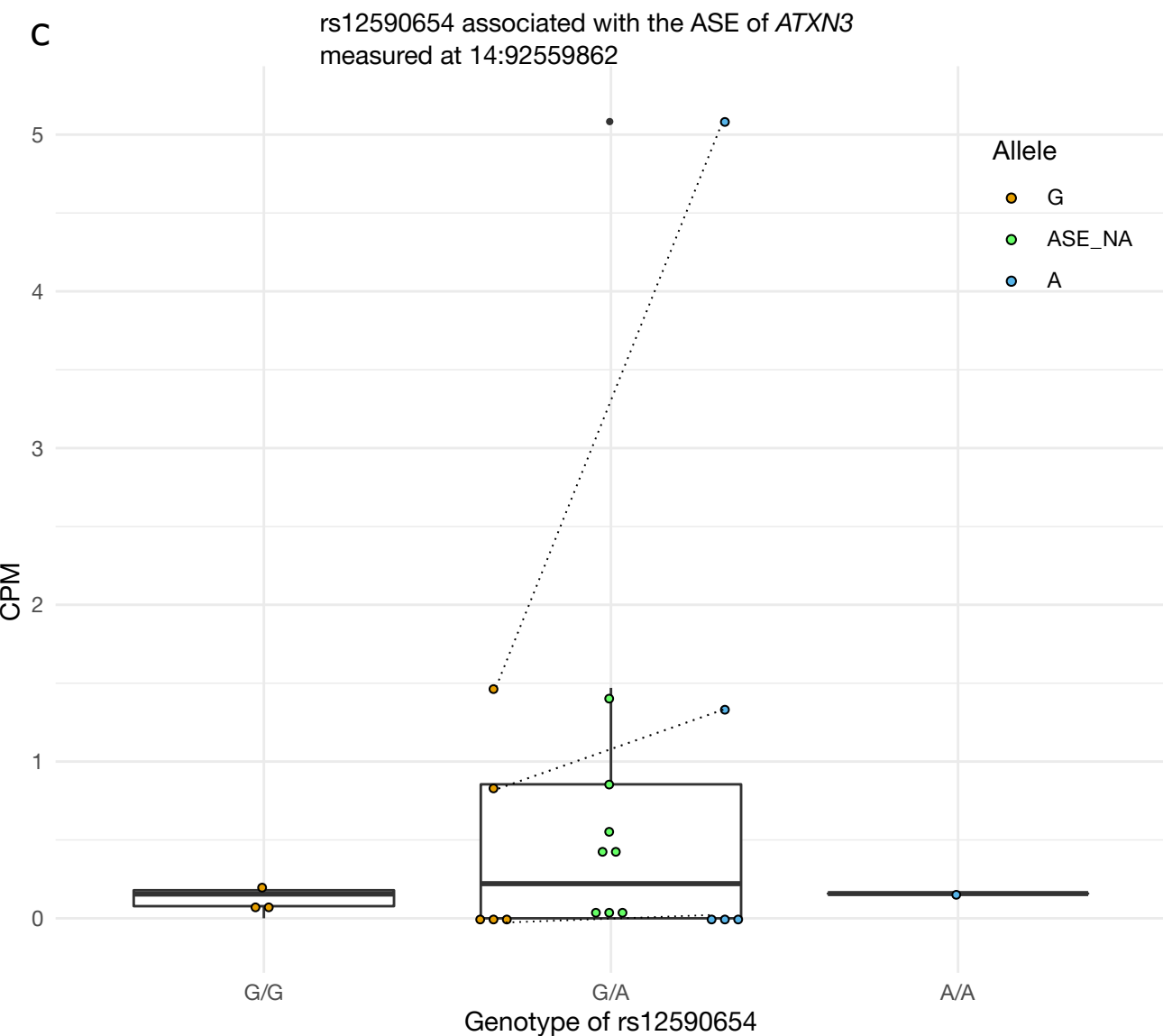

Supplement: Supplementary file 12 — Figure S2 [file 41398_2022_1913_MOESM12_ESM.pdf]

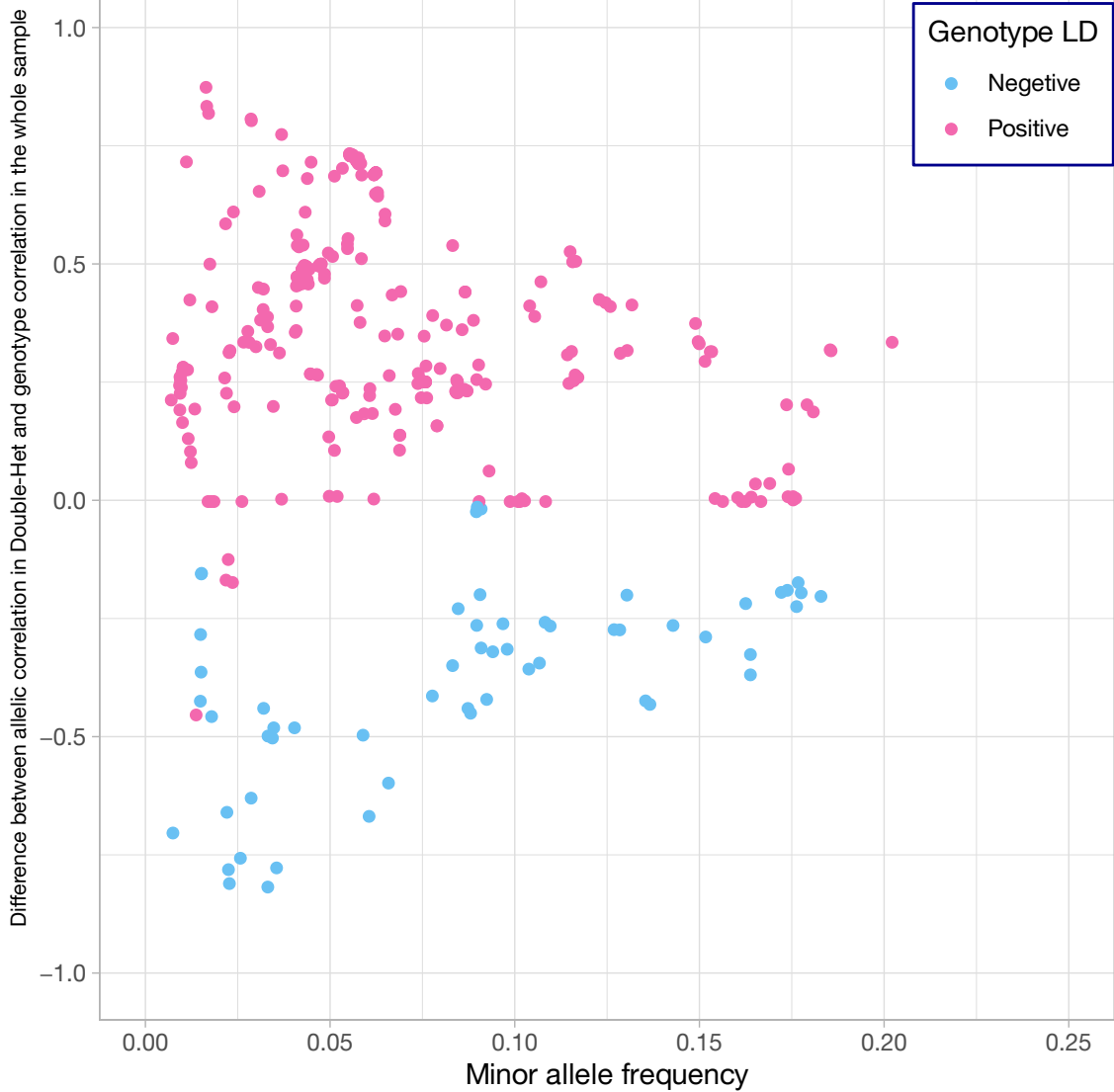

Supplement: Supplementary file 15 — Figure S5 [file 41398_2022_1913_MOESM15_ESM.pdf]

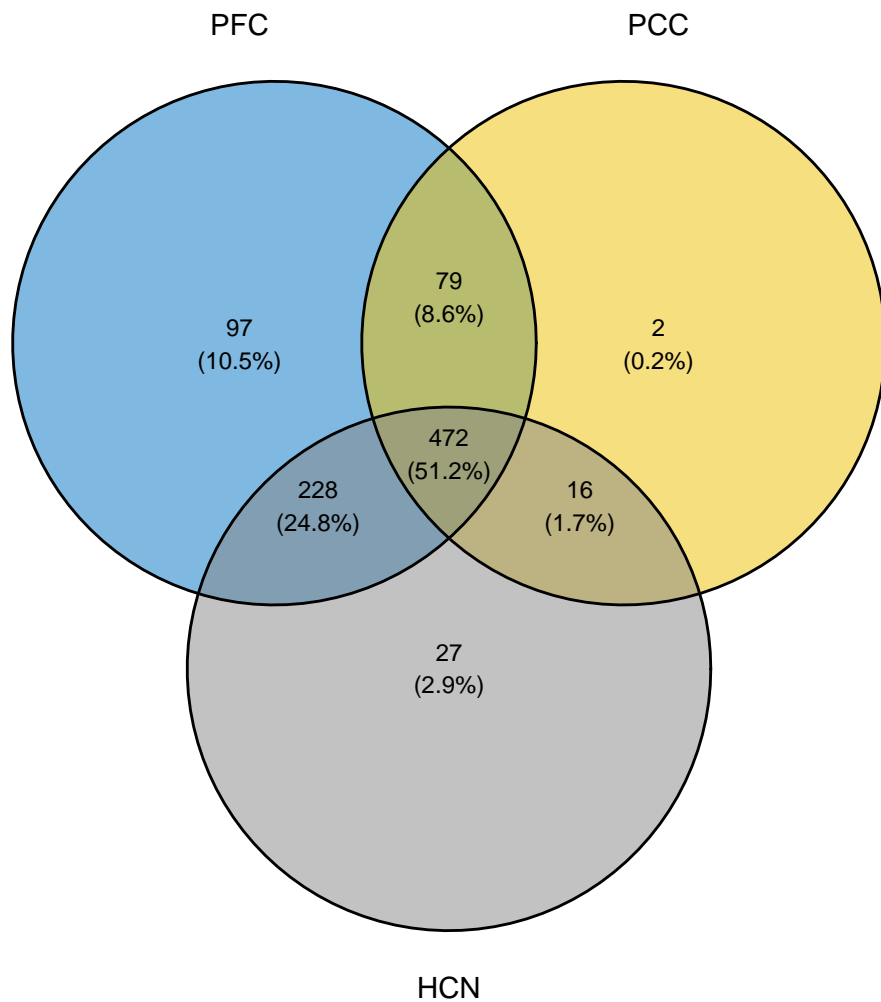

Supplement: Supplementary file 16 — Figure S6 [file 41398_2022_1913_MOESM16_ESM.pdf]

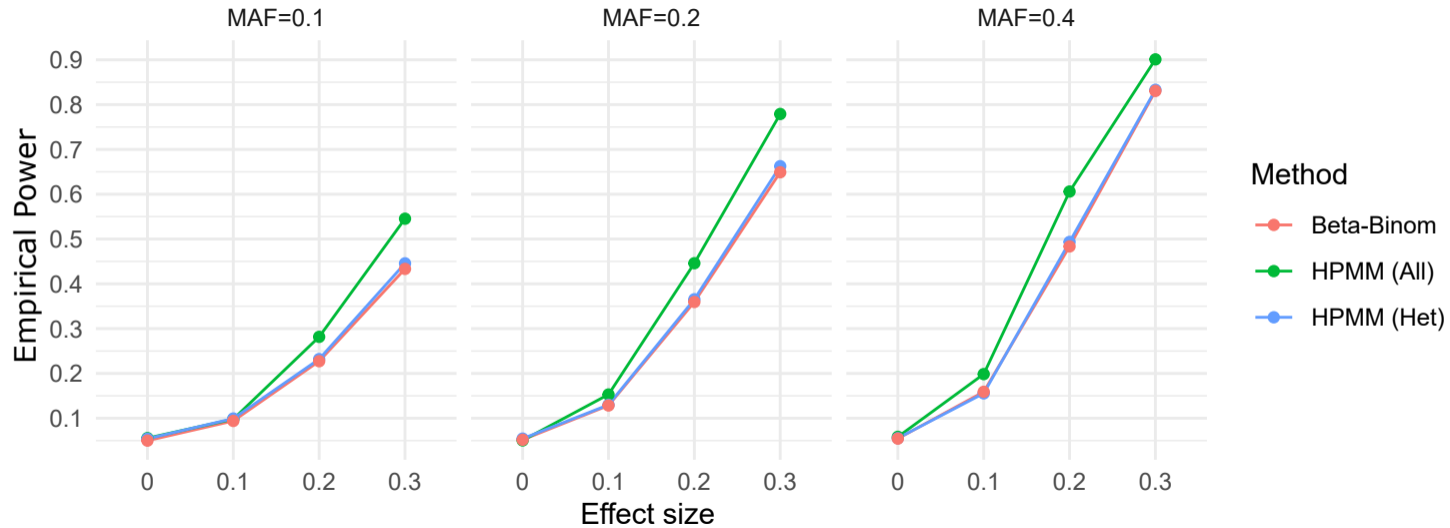

Supplement: Supplementary file 17 — Figure S7 [file 41398_2022_1913_MOESM17_ESM.pdf]
