# Supplementary material for: Allele-specific analysis reveals exon- and cell-type-specific regulatory effects of Alzheimer’s disease-associated genetic variants
Source: Transl Psychiatry. 2022 Apr 18;12:163. doi: 10.1038/s41398-022-01913-1 (PMC9016079; doi:10.1038/s41398-022-01913-1)
Supplement: Supplementary file 13 — Figure S3 [file 41398_2022_1913_MOESM13_ESM.pdf]

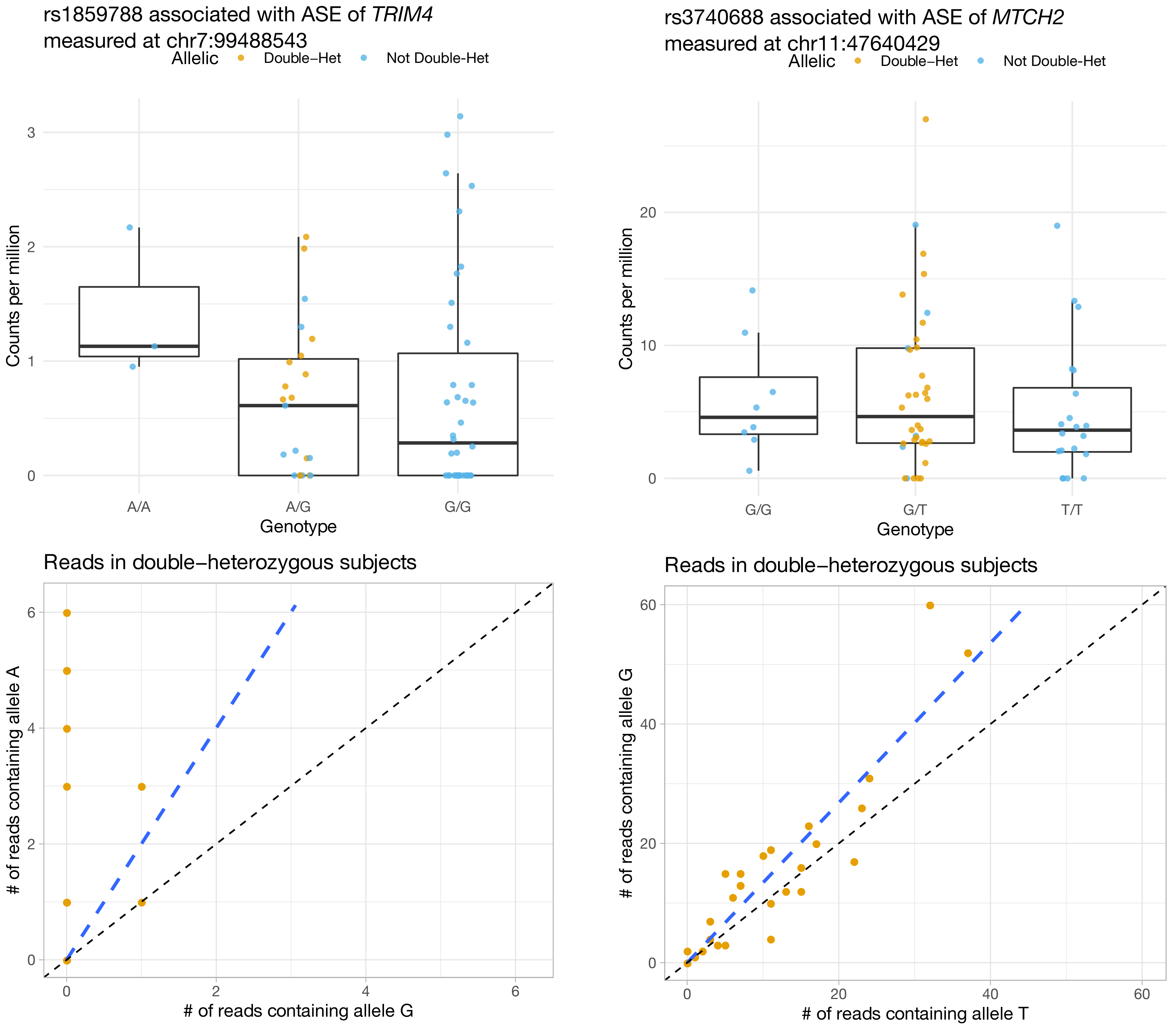
rs1859788 associated with ASE of *TRIM4*
measured at chr7:99488543
Allelic
Double−Het
Not Double−Het
Counts per million
0
1
2
3
A/A
A/G
G/G
Genotype
rs3740688 associated with ASE of *MTCH2*
measured at chr11:47640429
Allelic
Double−Het
Not Double−Het
Counts per million
0
10
20
G/G
G/T
T/T
Genotype
Reads in double−heterozygous subjects
# of reads containing allele A
0
2
4
6
# of reads containing allele G
Reads in double−heterozygous subjects
# of reads containing allele G
0
20
40
60
# of reads containing allele T
