# Supplementary material for: Allele-specific analysis reveals exon- and cell-type-specific regulatory effects of Alzheimer’s disease-associated genetic variants
Source: Transl Psychiatry. 2022 Apr 18;12:163. doi: 10.1038/s41398-022-01913-1 (PMC9016079; doi:10.1038/s41398-022-01913-1)
Supplement: Supplementary file 14 — Figure S4 [file 41398_2022_1913_MOESM14_ESM.pdf]

a Coding strategy for testing allelic imbalance for an exonic SNP of which the ASE is measured

Genotype-level data set

Subject ID	Genotype	Dosage	Count	Library
1	A/A	0	5	1000
2	A/a	1	14	1000
3	a/a	2	10	1000

Allelic expression available for the heterozygous subject

Allele-specific data set

Subject ID	Allele	Allele coding	Count	Library
1	A	0	5	1000
2	A	0	5	500
2	a	1	9	500
3	a	1	10	1000

b Coding strategy for testing allelic imbalance for a GWAS SNP through the haplotype with an exonic SNP

Subject ID	GWAS SNP	Exonic SNP	Count	Library
1	A/A	B/b	5	1000
2	A/a	B/b	12	1000
3	A/a	B/B	9	1000
4	a/a	B/B	10	1000

Allelic expression available for the double-heterozygous subject

Subject ID	GWAS Allele	GWAS coding	Count	Library
1	A	0	5	1000
2	A	0	5	500
2	a	1	7	500
3	A/a	0.5	9	1000
4	a	1	10	1000
